# Partial ischemia as molecular medicine: molecular mechanisms and clinical horizons of blood flow restriction training

**DOI:** 10.3389/fphys.2026.1877336

**Published:** 2026-08-05

**Authors:** Sirui Wang, Taiwei Guo, Yating Zhang

**Affiliations:** 1 College of Physical Education, Yangzhou University, Yangzhou, Jiangsu, China; 2 Sports Coaching College, Beijing Sport University, Beijing, China

**Keywords:** angiogenesis, blood flow restriction, cardiovascular adaptation, growth hormone, hypoxia-inducible factor-1α, kaatsu training, metabolic stress, mTORC1 signaling

## Abstract

**Background:**

Blood flow restriction (BFR) exercise combines low-intensity contractions with partial arterial inflow restriction and venous occlusion to generate a localized hypoxic, metabolite-rich milieu that activates a broad spectrum of molecular pathways.

**Aim:**

To synthesize contemporary (2020–2026) evidence on the systemic molecular effects of BFR across skeletal-muscle, endocrine, cardiovascular, immune, and neuromuscular systems, and to connect these mechanisms to clinical practice.

**Approach:**

Narrative review supported by a structured PubMed/Web of Science/Scopus search prioritizing randomized trials and recent systematic reviews and meta-analyses.

**Key findings:**

BFR engages mTORC1 signaling, myostatin suppression, satellite-cell proliferation, HIF-1α stabilization, and angiogenic gene expression to produce hypertrophic and functional outcomes broadly comparable to high-load resistance training; it elicits acute endocrine (growth hormone, IGF-1, testosterone, catecholamines) and metabolic (lactate, reactive oxygen species, AMPK) responses, together with cardiovascular, immune, and neural adaptations whose systemic clinical magnitude is modest and population-dependent. It is worth noting that the hypertrophic and signaling effects described arise from low-load contraction performed under occlusion; neither section implies an anabolic effect of passive occlusion without contraction.

**Conclusion:**

BFR is a mechanistically distinct and broadly applicable modality whose clinical value depends on rigorous individualization, careful safety stratification, and continued mechanistic investigation.

## 1 Introduction

Blood flow restriction (BFR) exercise, also referred to historically as KAATSU training or vascular occlusion training, has evolved from a niche laboratory curiosity into one of the most extensively studied and clinically translated exercise modalities of the twenty-first century. Conceptualized in Japan in the late 1960s and refined by Sato in the 1970s, the technique relies on the controlled application of an external pneumatic cuff to the proximal portion of a limb to partially restrict arterial inflow while substantially impeding venous outflow during exercise (Hurr, 2025; He et al., 2025). The resulting localized hypoxia, metabolite accumulation, and cellular stress allow contractions performed at as little as 20–30% of one-repetition maximum to elicit muscular and systemic responses comparable to those typically requiring loads greater than 65–85% of one-repetition maximum. This counterintuitive finding has fundamentally reshaped exercise prescription philosophies for clinical rehabilitation, geriatric care, and elite sport performance. The growing volume of investigations, particularly those published in 2025 and 2026, reflects both the technique’s expanding scope and an increasingly nuanced appreciation for its molecular underpinnings (Su et al., 2025; Cao et al., 2026; Kataoka et al., 2026). Importantly, what was once viewed primarily as a peripheral muscle stimulus is now understood to elicit systemic biological responses encompassing endocrine, cardiovascular, immune, and neural systems. Beyond peripheral and endocrine systems, BFR exercise also engages the central nervous system: in addition to motor-unit and pain-modulatory effects, exercise-induced increases in brain-derived neurotrophic factor (BDNF) have been reported with conventional exercise and are hypothesized to accompany the metaboreflex and hypoalgesic responses to BFR. Such a broad mechanistic footprint warrants a comprehensive review that synthesizes the latest molecular evidence. The present review therefore aims to integrate emerging mechanistic insights with clinically meaningful adaptations to clarify both the promise and the precautions of BFR exercise across diverse populations. By doing so, this work hopes to bridge the gap between bench-side molecular biology and bedside clinical application.

The conceptual appeal of BFR emerges from its ability to partially dissociate mechanical load from biological stimulus, a phenomenon that has prompted vigorous investigation into the convergence of mechanical and metabolic signaling within skeletal muscle. Recent scoping work suggests that BFR engages a constellation of molecular modulators including the mechanistic target of rapamycin complex 1, myostatin, hypoxia-inducible factor-1 alpha, vascular endothelial growth factor, and a host of myokines through pathways that are partially overlapping with, yet mechanistically distinct from, traditional resistance training (He et al., 2025). This molecular signature has been linked not only to muscle protein synthesis and hypertrophy but also to favorable systemic cardiovascular and immune adaptations, raising the possibility that BFR may exert benefits beyond the trained limb. Findings from recent meta-analyses confirm that BFR resistance training produces comparable gains in muscle cross-sectional area and one-repetition maximum strength when contrasted with high-load training, while imposing markedly lower joint and connective tissue stress (Jing et al., 2024; Su et al., 2025). For populations who cannot tolerate high mechanical loads, including post-surgical patients, frail older adults, and those with chronic disease, this distinction is clinically transformative. Furthermore, evidence accumulating over the past two years has begun to clarify how dosing parameters cuff width, applied pressure relative to limb occlusion pressure, exercise volume, and rest interval shape the magnitude and quality of the molecular response (Ren et al., 2025; Lin et al., 2025). At the same time, concerns regarding cardiovascular safety, particularly the engagement of the exercise pressor reflex and metaboreflex, remain incompletely resolved (Suggitt et al., 2024). These considerations underscore the need for a unified review that not only catalogs molecular effects but also contextualizes them within evolving safety frameworks. The rapid growth of the BFR literature also presents an opportunity to identify gaps and harmonize protocols across studies.

Equally compelling is the translational momentum BFR has generated across rehabilitative and clinical landscapes, with applications ranging from anterior cruciate ligament reconstruction to chronic obstructive pulmonary disease and intensive care unit-acquired weakness (Solie et al., 2023; Mathur et al., 2025; Alves et al., 2026). For example, in a single-subject case report (n = 1; Level IV evidence), Solie et al. (2023) followed a fifteen-year-old male soccer player who, after anterior cruciate ligament reconstruction with a bone–patellar tendon–bone autograft, completed a sixteen-week protocol integrating clinic- and home-based BFR; by the program’s end the surgical limb showed larger thigh girth and lean mass than the contralateral non-surgical limb (Solie et al., 2023). Because this is an uncontrolled single case, it is offered solely to illustrate clinical potential and not as generalizable evidence; the mechanistic conclusions of this review rest on the controlled studies and meta-analyses discussed in later sections. Such case examples reinforce the practical relevance of mechanistic findings drawn from controlled laboratory studies. In parallel, Mathur et al. demonstrated that a low-intensity BFR regimen could probe satellite cell function and modulate myostatin and MuRF1 transcript expression in critical illness survivors, offering a novel investigational tool to study persistent muscle wasting following intensive care discharge. On the contrary, several reviews have addressed BFR over the past five years. Patterson et al. (2019) established methodological and safety consensus; Cahalin et al. (2022) and Li et al. (2025) synthesized its role in sarcopenia; Loenneke and Hammert (2024) provided a twenty-five-year retrospective; and He et al. (2025) summarized resistance-training modes in athletic populations. These syntheses, however, have largely examined the muscular, endocrine, cardiovascular, immune, and neural consequences of BFR in isolation, and most predate the substantial body of primary work published in 2025 and 2026. The specific contribution of the present review is therefore threefold: (i) it integrates these traditionally siloed systems into a single cross-system molecular narrative; (ii) it incorporates the most recent 2025–2026 trials and meta-analyses not yet available to earlier syntheses; and (iii) it explicitly links mechanism to clinical decision-making through illustrative cases and a structured safety framework. We do not claim novelty for the individual mechanisms themselves, which are well established, but for their systematic integration across physiological systems.

## 2 Methodological foundations and application parameters

### 2.1 Study design and reporting framework

The present work was conducted as a structured narrative review with elements of a scoping synthesis, designed to integrate mechanistic, physiological, and clinical evidence regarding the systemic molecular effects of blood flow restriction (BFR) exercise. A detailed methodology was selected because the breadth of the research question spans multiple domains including molecular signaling, endocrine biology, cardiovascular physiology, inflammation, neuromuscular adaptation, and clinical translation that would be inadequately captured by a single quantitative meta-analytic framework. Where appropriate, the review draws on quantitative evidence summarized in existing systematic reviews and meta-analyses, but it does not pool effect sizes across primary studies. The reporting structure was informed by the Preferred Reporting Items for Systematic Reviews and Meta-Analyses Extension for Scoping Reviews (PRISMA-ScR) and by general best-practice guidance for narrative biomedical reviews. This hybrid framework permits both breadth and methodological transparency, providing readers with a clear audit trail of how the evidence base was identified, screened, and synthesized. The review protocol was developed *a priori* and refined through iterative consultation with the underlying research question, with explicit attention to the temporal scope, source typology, and thematic coverage. No formal protocol was registered in PROSPERO because the work is a narrative integration rather than a traditional systematic review, although the search strategy was documented in advance. Quality appraisal of included evidence was performed informally, with greater weight assigned to randomized controlled trials, systematic reviews, and mechanistic primary studies than to single-arm observational reports. The reporting decisions described in this section are intended to make the review reproducible at the level of search strategy, selection criteria, and thematic synthesis. The overall aim was to balance comprehensiveness with critical appraisal, prioritizing recent high-quality evidence while incorporating foundational mechanistic studies where relevant. By documenting the methodology transparently, this work seeks to support readers in interpreting both the strength of its conclusions and the boundaries of its evidence base.

### 2.2 Databases and information sources

A systematic search of the published biomedical literature was conducted across multiple complementary electronic databases to ensure broad coverage of mechanistic, clinical, and applied research on blood flow restriction exercise. The primary databases queried were PubMed/MEDLINE, Web of Science, Scopus, and SPORTDiscus, which together provide robust coverage of physiological, clinical, and sport science literature. Supplementary searches were performed in Embase, the Cochrane Central Register of Controlled Trials, and CINAHL to capture additional clinical trial and rehabilitation literature. Google Scholar was used as a secondary tool to identify grey literature, conference proceedings, and any recent publications not yet fully indexed in primary databases. Hand-searching of reference lists in key systematic reviews and recent narrative reviews was conducted to identify additional studies missed by database searches, with particular attention to landmark mechanistic studies. The ClinicalTrials.gov registry was consulted to identify ongoing trials and recently completed work whose results may have been published in preprint or early-access form. Frontiers in Physiology, the Journal of Applied Physiology, Sports Medicine, Medicine & Science in Sports & Exercise, the European Journal of Applied Physiology, and the Journal of Strength and Conditioning Research were also browsed directly for the most recent issues, as these journals publish a substantial proportion of contemporary BFR research. The search was conducted between January and April 2026, with a final update search performed in April 2026 to ensure inclusion of the most recent literature. No language restrictions were applied during the initial search, although only English-language full-text articles were ultimately included in the final synthesis. The decision to use multiple databases was based on evidence that single-database searches systematically miss relevant studies in interdisciplinary fields such as BFR research.

Studies were considered eligible for inclusion if they met several predetermined criteria designed to ensure relevance, methodological adequacy, and alignment with the review’s mechanistic and translational aims (**Table 1**). First, studies were required to investigate the effects of blood flow restriction exercise as an experimental intervention or as a primary focus of the analysis, regardless of whether it was applied alone or in combination with other modalities such as resistance training, aerobic exercise, neuromuscular electrical stimulation, or whole-body vibration. Second, eligible studies were required to report on at least one of the following: molecular or cellular signaling outcomes (e.g., mTORC1, myostatin, HIF-1α, VEGF, satellite cells), hormonal or endocrine responses, metabolic or bioenergetic markers, redox biology, cardiovascular or hemodynamic parameters, inflammatory or immune markers, neuromuscular activation, or functional/clinical outcomes such as strength, hypertrophy, or rehabilitation milestones. Third, studies conducted in human participants were prioritized, although animal and *in vitro* studies were retained when they provided unique mechanistic insight that could not be obtained from human work. Fourth, randomized controlled trials, controlled clinical trials, crossover studies, prospective cohort studies, single-arm interventional studies, and mechanistic biopsy studies were all eligible, reflecting the diverse methodological landscape of BFR research. Fifth, systematic reviews, meta-analyses, scoping reviews, and narrative reviews published from 2020 onward were included as secondary sources to identify primary literature and to anchor synthesis. Sixth, case studies and case series were eligible specifically when they provided illustrative translational examples that bridged mechanistic and clinical findings, as exemplified by the case reports of post-surgical and critical illness patients integrated throughout the review. Seventh, articles published in peer-reviewed journals indexed in major biomedical databases were eligible. Eighth, English-language full-text availability was required for final inclusion in the synthesis. Together, these criteria reflect a deliberate balance between scientific rigor and the breadth required for a comprehensive mechanistic and translational review.

**Table 1:** Summary of inclusion and exclusion criteria.

Domain	Inclusion criteria	Exclusion criteria
Intervention	BFR as primary or significant intervention	BFR not a primary focus; pathological ischemia models without exercise
Outcomes	Molecular signaling, hormonal, metabolic, redox, cardiovascular, immune, neuromuscular, functional/clinical	No relevant physiological or clinical outcome reported
Study Design	RCT, controlled trial, crossover, prospective cohort, mechanistic biopsy study, review, illustrative case study	Editorial, commentary, conference abstract only, duplicate, retracted
Population	Human participants (priority); animal/*in vitro* for unique mechanistic insight	Veterinary or non-mammalian applications
Publication Period	2020–2026 (priority); foundational studies retained selectively for mechanistic context	Pre-2020 work superseded by recent evidence
Language/Access	English-language, peer-reviewed, full-text accessible	Non-English without translation; full text unavailable

Articles were excluded if they failed to meet the inclusion criteria or if they presented specific limitations that compromised their interpretability or relevance for the review. Articles were excluded if blood flow restriction was not a primary or significant focus of the investigation, including studies in which BFR was mentioned only incidentally or as a peripheral comparator. Studies in which the occlusion stimulus was applied without exercise or without sufficient mechanistic or physiological characterization were excluded unless they specifically informed safety or methodological discussions. Articles examining pathological ischemia (such as in stroke, peripheral artery disease pathophysiology, or critical limb ischemia) without an exercise component were excluded, as the review focused on BFR as an exercise modality rather than as a model of disease. Articles whose full text could not be obtained through institutional access, interlibrary loan, or direct author contact were excluded. Editorials, letters to the editor, commentaries without substantial primary data or synthesis, and conference abstracts without subsequent full publication were generally excluded, although select commentaries from leading experts were retained when they provided substantive critical perspective. Duplicate publications, retracted articles, and articles flagged by integrity databases were excluded. Articles in languages other than English without available translation were excluded at the final synthesis stage, although their abstracts were considered during the initial screening. Studies of veterinary or non-mammalian BFR applications were excluded as they fell outside the scope of human and translationally relevant mechanistic research. Lastly, articles whose methodology was deemed insufficiently described to permit basic interpretation were excluded, although this judgment was applied conservatively to avoid inappropriate exclusion of valuable mechanistic work.

### 2.3 Cuff design and pressure determination

**Figure 1:**
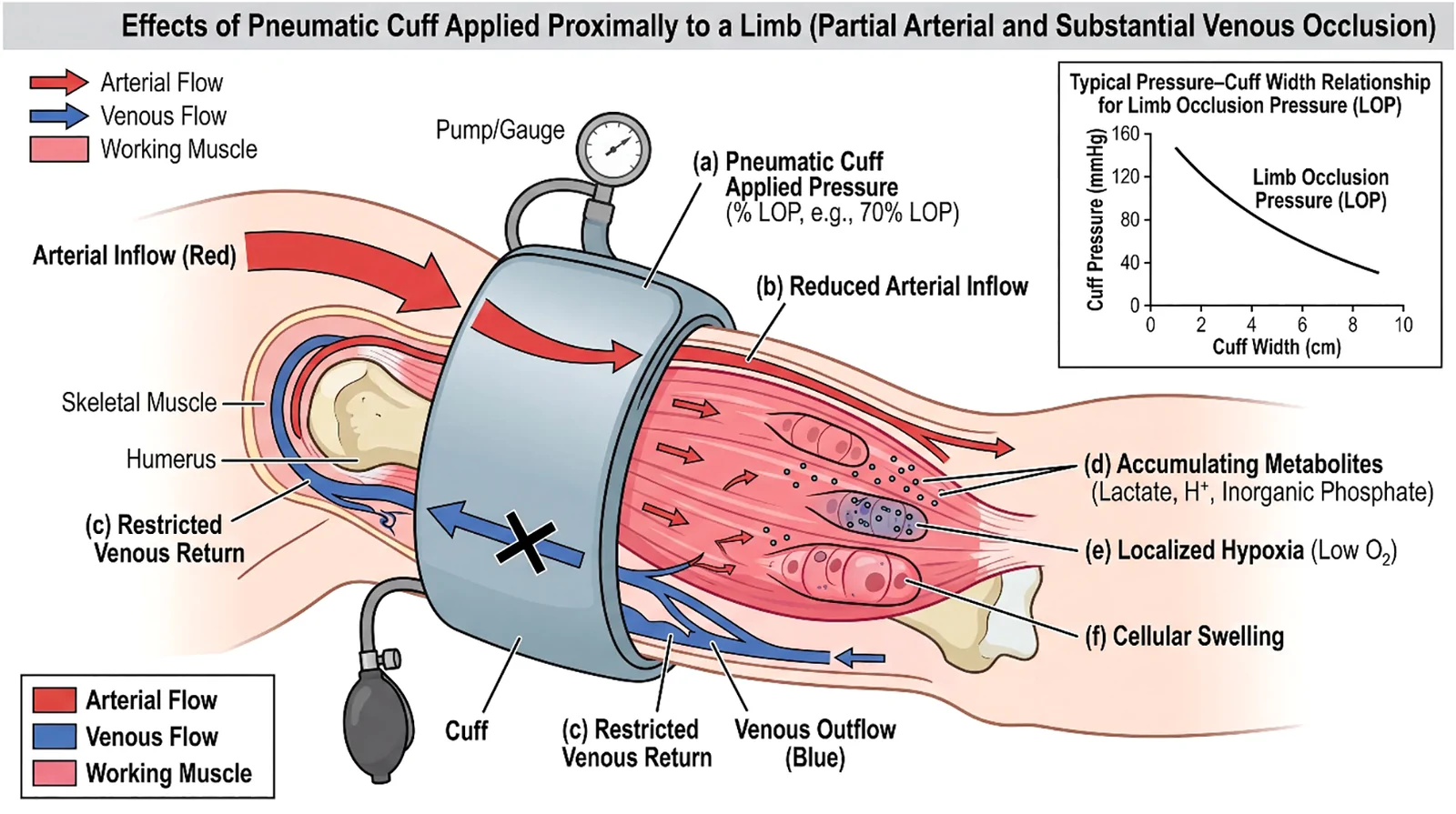
Schematic representation of the BFR mechanism. This figure depicts a cross-section of a limb with a pneumatic cuff applied proximally, illustrating the partial arterial inflow restriction (shown as a narrowed arrow toward the muscle) and the substantial venous outflow occlusion (shown as a blocked or markedly narrowed return flow).

The methodological backbone of any BFR application rests on the selection of an appropriate cuff and the precise determination of occlusion pressure, two variables that jointly govern both efficacy and safety (**Figure 1**). Throughout this review, it should be noted that the studies synthesized differ considerably in cuff characteristics, pressure-prescription method, exercise mode, and population; consequently, the mechanistic and clinical conclusions drawn below describe the weight and direction of current evidence rather than precisely transferable effect sizes. Modern cuffs are typically pneumatic and range in width from 5 to 18 centimeters; wider cuffs require lower absolute pressures to achieve a given degree of arterial restriction owing to their broader contact surface (de Queiros et al., 2024). Personalized prescription is now considered the gold standard, with most contemporary protocols expressing applied pressure as a fraction of the individual’s limb occlusion pressure, typically 40–50% of limb occlusion pressure for the upper limbs and 60–80% for the lower limbs, consistent with current consensus recommendations (Patterson et al., 2019). Limb occlusion pressure itself reflects the minimum cuff pressure required to fully arrest arterial inflow at rest and is influenced by limb circumference, blood pressure, body composition, and cuff geometry. Failure to individualize pressure can result in either insufficient stimulus or unsafe overload, particularly in older adults and clinical populations whose vascular reactivity differs from that of young healthy participants (Ren et al., 2025). Recent work has emphasized that even the position in which limb occlusion pressure is measured for instance, supine versus the exercise position itself affects the absolute value obtained, prompting calls for standardized assessment protocols (de Queiros et al., 2024). Importantly, limb occlusion pressure is position-dependent: it changes with body posture (supine versus seated or standing), limb elevation, and joint angle, because these alter hydrostatic pressure and arterial inflow. A pressure measured supine may therefore under- or over-estimate the true occlusion stimulus during seated or standing exercise. For standardization and reproducibility, we recommend that LOP be determined in the same position and joint configuration in which the BFR exercise will subsequently be performed, and that the measurement position be explicitly reported. Failure to do so is a likely contributor to the between-study heterogeneity discussed in Section 12.1. Furthermore, lower-pressure protocols (40–50% of limb occlusion pressure) have been associated with comparable hypertrophic outcomes but with substantially reduced cardiovascular strain, making them attractive for at-risk populations. Importantly, the application of practical BFR using elastic non-pneumatic cuffs that approximate but do not directly measure arterial occlusion has been validated for field-based and home-based use, although it sacrifices precision for accessibility (Solie et al., 2023). The contemporary literature thus reflects a tension between rigor and feasibility, with optimal practice depending on context, goals, and patient profile. Properly calibrated, however, BFR pressure becomes a powerful dosing variable rather than a one-size-fits-all manipulation.

In physically inactive young adults, adding BFR to a matched low-load multi-component Programme produced modest additional gains in muscle thickness and selected functional outcomes relative to the same Programme without occlusion, reinforcing the value of BFR as an adjuvant to, rather than a replacement for active training (Ahmad et al., 2026). Pressure interacts with cuff width, limb circumference, and underlying tissue composition in ways that have only recently been formally modeled, and these relationships have important implications for both research interpretation and clinical practice. Mouser et al. demonstrated that arterial occlusion is achieved at lower pressures with wider cuffs and on smaller limbs, a phenomenon especially relevant in pediatric, geriatric, and amputee populations (Mouser et al., 2019). Conversely, narrow cuffs require higher absolute pressures to achieve occlusion and concentrate compressive force over neurovascular bundles, increasing the risk of paraesthesia or nerve irritation; for this reason, wider, properly fitted pneumatic cuffs are preferred over narrow cuffs or thin elastic bands in clinical practice. Higher pressures generally elicit greater accumulation of metabolites and more pronounced anabolic hormonal responses, but they also amplify hemodynamic perturbations and the activation of the sympathetic nervous system (Bane et al., 2024; Thompson et al., 2024). A practical illustration of these tradeoffs comes from work by Li et al., who showed that resistance exercise performed at 70% of arterial occlusion pressure produced substantially higher growth hormone and testosterone responses than the same exercise at 40% of arterial occlusion pressure, but at the cost of greater perceived exertion and discomfort (Li et al., 2021). Such findings argue against the simplistic notion that more pressure is uniformly better; rather, optimal pressure depends on the desired outcome, training history, and tolerance of the individual. The current consensus suggests that pressures between 40% and 80% of limb occlusion pressure encompass an effective and reasonably safe range for most healthy populations. For clinical populations, however, lower pressures and shorter exposure durations are typically warranted as a starting point. As Cao et al. recently reported in older adults with tibial plateau fractures, even short-term, conservatively dosed BFR protocols can yield meaningful improvements in knee function and quality of life (Cao et al., 2026). Continued refinement of dose-response relationships across populations and outcomes therefore remains a research priority. Beyond expressing pressure as a percentage of limb occlusion pressure, surrogate physiological targets have been proposed to titrate or verify the occlusion stimulus, for example, achieving a target reduction in muscle oxygen saturation measured by near-infrared spectroscopy, or a defined exercise heart-rate rise. These approaches are attractive because they reflect the actual tissue-level effect of occlusion rather than an externally applied pressure; however, they are not yet standardized or validated against hypertrophic outcomes and currently complement, rather than replace, LOP-based prescription (de Queiros et al., 2024).

### 2.4 Exercise protocols and loading strategies

Beyond pressure considerations, the design of the exercise protocol itself critically shapes the molecular signal generated by BFR, and several discrete loading paradigms have emerged within the contemporary literature. Among the many published BFR configurations, some parameters are now relatively well supported, whereas others remain emerging (**Table 2**). The best-supported resistance-exercise template is a low load (approximately 20–30% of one-repetition maximum) performed for 75 repetitions divided as 30/15/15/15 across four sets with short (~30–45 s) inter-set rest, using cuff pressures individualized as a percentage of limb occlusion pressure (commonly ~40–50% LOP for the upper limb and ~60–80% LOP for the lower limb) rather than fixed absolute pressures (Patterson et al., 2019). Emerging or less-thoroughly validated approaches, including practical/elastic-cuff BFR, walking and other aerobic BFR modes, passive or intermittent occlusion, and very-high-pressure low-volume schemes show promise but rest on smaller or more heterogeneous evidence bases and warrant cautious prescription. For a current methodological overview that situates these variants relative to one another, see Scott et al. (2023) (Patterson et al., 2019; He et al., 2025). This pattern maximizes metabolic stress through repeated bouts of contraction in an ischemic environment and is the protocol most consistently associated with hypertrophic and strength outcomes. Aerobic BFR protocols, in contrast, typically employ low-to-moderate intensity treadmill walking or cycling with intermittent or continuous cuff inflation, eliciting improvements in aerobic capacity and muscular endurance even when the absolute aerobic stimulus is modest (Chen et al., 2025). Combined paradigms, such as concurrent BFR resistance training periodized with traditional high-load training, have recently attracted attention for their ability to potentiate adaptations through complementary mechano-metabolic pathways (He et al., 2025). Newer applications also include neuromuscular electrical stimulation combined with BFR, which generates contraction-like stimuli in patients unable to exercise volitionally; combining BFR with electrical stimulation has been shown to attenuate disuse muscle atrophy and support anabolic signaling (Slysz et al., 2021;. Mangahas et al., 2026; Yang et al., 2024). Each protocol carries distinct implications for which molecular pathways are engaged most prominently. Practitioners must therefore align protocol selection with both the target adaptation and the patient’s physiological context.

**Table 2:** Common BFR exercise parameters across populations and goals.

Population/goal	Pressure (% LOP)	Load (% 1RM)	Volume (reps × sets)	Frequency	Key source(s)
Healthy adult hypertrophy	40–50% UL/60–80% LL	20–30%	30/15/15/15 × 4 sets	2–4×/week	Patterson et al. (2019); He et al. (2025)
Athletic strength/power	40–50% UL/60–80% LL	20–40% (or combined HL)	3–5 sets × 8–15 reps	2–3×/week	Su et al. (2025); He et al. (2025)
Older adult/sarcopenia	40–60%	20–30%	30/15/15/15 × 4 sets	2–3×/week	Ren et al. (2025); Li et al. (2025)
Post-surgical (e.g., ACLR)	60–80%	20–30%	30/15/15/15 × 4 sets	2–3×/week clinic; optional daily low-load practical BFR (home)	Solie et al. (2023); Butt and Ahmed (2024)
Critically ill/ICU survivor	40–50%	≤20% (or NMES)	Reduced (e.g., 2–3 sets)	2–3×/week	Mathur et al. (2025); Slysz et al. (2021)
Aerobic capacity (BFR walking/cycling)	40–60%	40–60% VO2max	5 × 3 min bouts	3×/week	Chen et al. (2025)

LOP, limb occlusion pressure; UL, upper limb; LL, lower limb; 1RM, one-repetition maximum; HL, high-load; NMES, neuromuscular electrical stimulation; ACLR, anterior cruciate ligament reconstruction. The post-surgical clinic frequency has been reconciled to 2–3×/week, with optional daily low-load practical BFR at home flagged as a distinct, lower-intensity home practice.

The interaction between BFR protocol parameters and patient-specific factors is a central theme of recent dosing literature, with several systematic reviews refining recommendations published in earlier years. For example, meta-analyses comparing low-load BFR with low-load training without occlusion report small-to-moderate standardized mean differences favoring BFR for hypertrophy and strength, but typically with wide confidence intervals and moderate-to-high statistical heterogeneity, indicating low-to-moderate certainty of evidence (Ren et al., 2025). Comparisons between low-load BFR and high-load training more often yield confidence intervals that include no meaningful difference, supporting equivalence in specific populations rather than superiority. Similarly, Su et al. demonstrated in a meta-analysis published in 2025 that combining BFR with high-load training in athletes yielded particularly large gains in maximal strength and power, illustrating BFR’s flexibility as either a primary or adjunct stimulus (Su et al., 2025). The case of a fifteen-year-old soccer player previously cited illustrates these principles in practice: his clinic-based BFR protocol used 80% of limb occlusion pressure with low-load knee extension and bilateral leg press exercises, and the addition of home-based practical BFR using elastic cuffs allowed for daily exposure that ultimately reversed thigh atrophy (Solie et al., 2023). On the opposite end of the clinical spectrum, Mathur et al. used a deliberately conservative four-week, low-intensity protocol with 50% limb occlusion pressure to safely probe muscle regeneration in critically ill survivors (Mathur et al., 2025). Together, these examples underscore that optimal protocol design is fundamentally context-dependent and must integrate physiological, mechanical, and patient-centered considerations. As BFR continues to mature as a clinical tool, increasingly sophisticated protocols may, in the future, leverage real-time physiological feedback such as near-infrared spectroscopy and electromyographic monitoring. We emphasize that this is a promising but as-yet largely unvalidated direction: to our knowledge no standardized, published feedback-driven BFR protocol currently exists, and the concept is discussed here as a research priority rather than an established method. The promise of such individualized prescription is substantial, but it must be balanced against the practical realities of clinical implementation. Rest and reperfusion intervals are themselves an important protocol variable. In continuous BFR, the cuff remains inflated through the brief inter-set rests (e.g., 30 s); in intermittent BFR, the cuff is deflated between sets or bouts to permit reperfusion, which lowers cumulative metabolic and cardiovascular strain. In aerobic BFR, occlusive bouts (e.g., 5 × 3 min of walking or cycling) are typically separated by approximately 30–60 s of cuff-deflated rest or low-intensity non-occluded activity; the choice between continuous and intermittent occlusion should be matched to the population (intermittent for higher-risk or clinical groups) and the target adaptation. Future protocol development will benefit from harmonization across studies and clearer reporting standards.

Because BFR amplifies systemic stress responses notably catecholamine release and the exercise metaboreflex, it should be programmed deliberately within an athlete’s overall load rather than added indiscriminately. We suggest that practitioners (i) count BFR sessions toward total weekly training stress rather than treating them as “free” low-load work; (ii) limit higher-pressure, higher-volume BFR to roughly two to three sessions per week with adequate recovery; (iii) avoid stacking demanding BFR onto already high-stress periods (e.g., competition microcycles or heavy-load blocks) without compensatory recovery; and (iv) monitor for markers of non-functional overreaching such as persistent fatigue, performance decrement, sleep disturbance, and autonomic changes (for example, suppressed heart-rate variability). Used judiciously, BFR can substitute for a portion of high-load volume while reducing joint and connective-tissue stress, but the systemic stress it imposes is real and must be accounted for in periodization.

## 3 Molecular mechanisms driving skeletal muscle hypertrophy

### 3.1 Activation of mTORC1 signaling cascade

**Figure 2:**
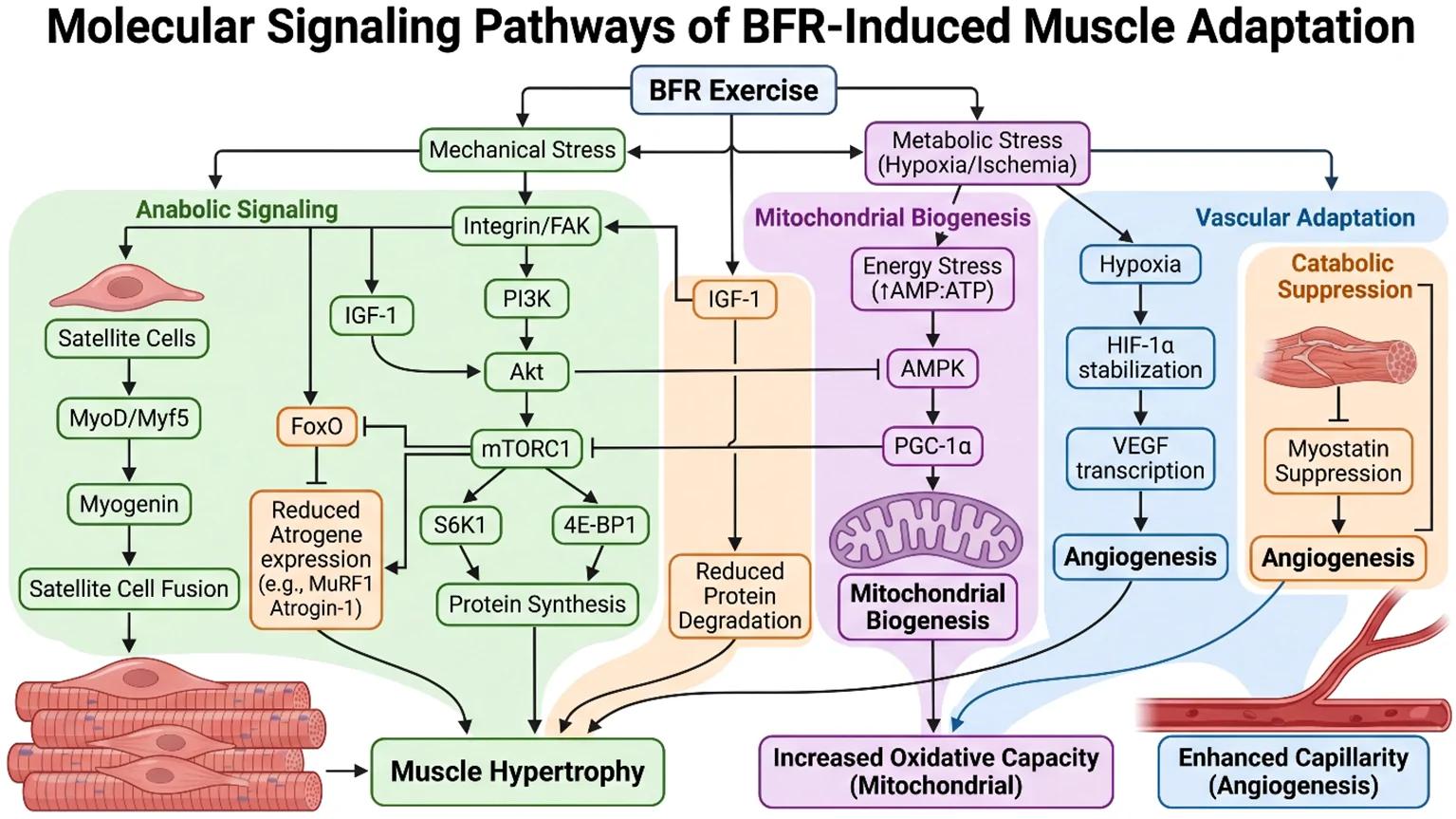
Integrated molecular signaling pathways activated by BFR exercise. This figure represents a comprehensive pathway diagram showing how BFR-induced metabolic stress and mechanical signaling converge on protein synthesis, mitochondrial biogenesis, and angiogenesis.

The mechanistic target of rapamycin complex 1, mTORC1, sits at the intersection of nutrient, energy, and mechanical signals to govern skeletal muscle protein synthesis, and it is widely accepted as a key node in BFR-induced hypertrophy. Following BFR exercise, phosphorylation of downstream effectors including ribosomal protein S6 kinase 1 (S6K1) and eukaryotic translation initiation factor 4E binding protein 1 (4E-BP1) increases markedly, signaling enhanced cap-dependent translation and ribosomal biogenesis (Fry et al., 2010; He et al., 2025). What makes BFR particularly intriguing is that this mTORC1 activation occurs at loads that, in the absence of cuff inflation, fail to elicit measurable protein synthesis or signaling activity (Fry et al., 2010; Gundermann et al., 2014). The dependence of this effect on mTORC1 was confirmed in a landmark study by Gundermann et al., who demonstrated that pretreatment with rapamycin abolished the BFR-induced increase in muscle protein synthesis. Subsequent investigations have shown that mTORC1 activation under BFR conditions involves both the canonical phosphatidylinositol 3-kinase/Akt pathway and emerging mechanotransduction pathways converging on Rheb and TSC1/TSC2 complexes (Pearson and Hussain, 2015; He et al., 2025). The role of metabolic stress in this activation cannot be overstated; lactate, hydrogen ion accumulation, and intracellular calcium signaling have each been implicated in the mTORC1 response to BFR (**Figure 2**). Recent rodent and human work also suggests that the mitogen-activated protein kinase pathway operates in parallel with mTORC1, providing a redundant signal for translational machinery (Natsume et al., 2018; He et al., 2025). The convergence of these mechano-metabolic inputs likely explains why BFR can match high-load training despite its modest mechanical signature. Together, these findings position mTORC1 as both a central mediator and a logical therapeutic target in BFR research. The longer-term trajectory of mTORC1 signaling is incompletely characterized. As with conventional resistance training, the acute signaling response tends to attenuate as training status improves (a repeated-bout effect), even while muscle cross-sectional area continues to increase over the first weeks of training before plateauing. Most molecular studies extend only to roughly 8–12 weeks, and serial biopsy data at 3–6 months or beyond are scarce; consequently, whether there is a true waning of the anabolic signal versus a maintained but smaller stimulus sufficient to preserve gains, remains an open question and a priority for longitudinal study.

Despite this strong mechanistic foundation, recent investigations have begun to nuance the simple view that mTORC1 activation alone explains the hypertrophic response to BFR. Fuchs et al. reported that in healthy young men subjected to two weeks of bed rest, daily BFR application without exercise did not preserve muscle mass or modulate daily protein synthesis rates, indicating that mechanical contraction is necessary to translate the metabolic stimulus into anabolic signaling (Fuchs et al., 2024). This finding suggests that BFR amplifies a contraction-dependent signal rather than independently activating mTORC1, refining our mechanistic understanding. In a complementary study, the same group reported that BFR did not affect satellite cell content or capillarization during prolonged unloading, further constraining the conditions under which BFR is effective (Fuchs et al., 2024). Fuchs et al. (2024) showed that, during two weeks of bed rest, daily BFR applied without muscle contraction neither preserved muscle mass nor raised daily protein synthesis, and did not affect satellite-cell content or capillarization. This finding constrains an important boundary condition: in humans, BFR appears to amplify a contraction-dependent anabolic signal rather than to independently switch on mTORC1. Practically, this means the occlusion stimulus is an adjuvant to active or evoked contraction (including neuromuscular electrical stimulation), not a passive substitute for it. Throughout this review, therefore, statements that BFR “activates” anabolic signaling should be read as referring to low-load contraction performed under occlusion, and claims of benefit in fully passive contexts are not supported by current human evidence. The mTORC1 pathway also displays time-dependent kinetics, with peak phosphorylation typically observed one to three hours post-exercise and waning over subsequent hours (Fry et al., 2010). Repeated exposure across training sessions appears necessary to translate acute signaling into measurable hypertrophy, with most studies showing meaningful muscle cross-sectional area increases after six to eight weeks of training (Jing et al., 2024). A particularly instructive case for this kinetic understanding is the rehabilitation of a fifteen-year-old soccer player who underwent anterior cruciate ligament reconstruction; daily home-based practical BFR exposure for sixteen weeks plausibly maintained tonic mTORC1 activation in concert with intermittent loading, ultimately reversing thigh atrophy (Solie et al., 2023). Such cases highlight that the mTORC1 response in clinical contexts is shaped by both the acute exercise stimulus and the cumulative training load. Continued mechanistic work is therefore needed to clarify how protocol parameters tune mTORC1 activation in real-world settings.

These mechanistic inferences should be interpreted cautiously. The acute anabolic-signaling data for BFR, including mTORC1 and p70S6K phosphorylation, come largely from a small number of muscle-biopsy studies with modest sample sizes, often in young, healthy men, and sampled at a limited number of post-exercise time-points. Acute increases in phosphorylation status are informative about candidate pathways but are imperfect surrogates for chronic hypertrophy, which depends on the cumulative integral of many such responses over weeks of training. The mechanisms described here should therefore be read as biologically plausible and partially evidenced rather than definitively established in humans.

### 3.2 Myostatin suppression and atrophy gene modulation

Equal in importance to anabolic activation is the suppression of catabolic and anti-anabolic pathways, and BFR exerts particularly notable effects on myostatin and the ubiquitin-proteasome system. Myostatin, a member of the transforming growth factor-beta superfamily, is widely recognized as a negative regulator of skeletal muscle growth, and its downregulation following BFR exercise is among the most consistent transcriptional findings in the field (Laurentino et al., 2012; Baig et al., 2022). Acute and chronic BFR exposures decrease myostatin mRNA expression in vastus lateralis biopsies, and these reductions are accompanied by parallel increases in growth and differentiation-associated serum protein-1 (GASP-1) and SMAD-7, both of which inhibit myostatin’s downstream signaling (Laurentino et al., 2012). Beyond myostatin itself, BFR has been shown to attenuate the expression of E3 ubiquitin ligases including muscle RING-finger protein-1 (MuRF1) and atrogin-1, which mark sarcomeric proteins for proteasomal degradation (Manini et al., 2011; Mathur et al., 2025). The integrated effect is a shift in the protein turnover balance toward net synthesis, even at low absolute training loads. Mathur et al. demonstrated this phenomenon strikingly in critical illness survivors with sustained muscle atrophy following intensive care unit-acquired weakness, showing that a brief BFR exercise regimen significantly altered MuRF1 transcript expression and reduced myostatin expression in trained versus untrained vastus lateralis (Mathur et al., 2025). Such findings support the use of BFR as a tool to interrogate the integrity of the satellite cell-myostatin-MuRF1 axis in vulnerable populations. The transcriptional reprogramming induced by BFR thus extends well beyond simple anabolic activation to encompass coordinated suppression of catabolic machinery.

The temporal profile of myostatin and atrogene suppression following BFR is increasingly understood to be a key determinant of long-term hypertrophic outcomes. Manini et al. showed that acute low-load resistance exercise with BFR significantly reduced myostatin mRNA within three hours, and this effect persisted for at least eight hours post-exercise (Manini et al., 2011). Repeated exposure across training cycles appears to entrench these transcriptional shifts, although interindividual variability remains substantial and is incompletely understood. Recent scoping work has highlighted that genetic variation in myostatin and other regulatory loci may explain why some individuals respond robustly to BFR while others remain non-responders, but no studies have yet directly linked specific polymorphisms to BFR adaptation (Herman et al., 2026). The interplay between myostatin and inflammatory cytokines is also receiving renewed attention; interleukin-6 and interleukin-15 have been shown to modulate myostatin expression and may amplify the catabolic suppression observed during BFR (Barawi et al., 2025). Furthermore, myostatin’s downstream effectors include not only protein synthesis machinery but also satellite cell quiescence regulators, suggesting that its suppression facilitates both hypertrophy and regenerative capacity. The case of the previously cited critically ill survivor cohort exemplifies the clinical relevance of these mechanisms; in patients whose underlying myopathy is partially driven by elevated myostatin signaling, BFR’s transcriptional reprogramming offers a therapeutic foothold (Mathur et al., 2025). Newer high-load studies that incorporate BFR also point to additive transcriptional effects, raising the possibility that periodized BFR-traditional combinations may maximize myostatin suppression (Su et al., 2025). This emerging picture casts BFR as a multifaceted modulator of the protein turnover balance rather than a simple anabolic stimulus. Continued mechanistic work will refine how clinicians can leverage these effects across populations. In addition, because myostatin is a secreted, circulating factor, its suppression after BFR could in principle exert systemic, endocrine-like effects on muscle outside the occluded limb, including proximal muscle groups. In practice, however, most measured reductions in myostatin expression are local to the exercised muscle, and direct human evidence for a remote anti-myostatin effect on untrained proximal muscle is currently lacking. We therefore treat any systemic anti-catabolic contribution as plausible but unproven.

### 3.3 Satellite cell proliferation and myogenic regulation

Skeletal muscle hypertrophy requires not only the synthesis of new contractile proteins but also the addition of new myonuclei to support enlarging fibers, and satellite cells provide this regenerative capacity. Although early BFR research focused on mTORC1 and protein synthesis, an emerging body of work demonstrates that BFR effectively activates satellite cell pools, leading to increases in satellite cell density and myonuclear addition (Nielsen et al., 2012; Mathur et al., 2025). Nielsen et al. first demonstrated that low-load BFR resistance training increased satellite cell content in human vastus lateralis to a degree comparable with high-load training, a finding that was once unexpected given the prevailing view that satellite cell activation required higher mechanical loads. When training load is matched to the individual’s clinical context, low-load BFR can produce hypertrophy and strength gains comparable to high-load resistance training, and may be preferable when high mechanical loads are contraindicated (e.g., early post-surgical rehabilitation, sarcopenia, or selected chronic-disease populations). It is not, on current evidence, uniformly superior to high-load training in healthy, load-tolerant individuals. Subsequent investigations have shown that satellite cell activation under BFR is accompanied by increased expression of myogenic regulatory factors including MyoD, Myf5, and myogenin, transcription factors that orchestrate satellite cell commitment, proliferation, and differentiation (Manini et al., 2011; Mathur et al., 2025). The acute upregulation of p21, a marker of satellite cell activation, has been documented within hours of low-load BFR exercise, supporting rapid mobilization of these stem-like cells. The integration of new myonuclei into existing fibers is essential for sustained hypertrophy, particularly in older adults whose satellite cell pools are typically depleted (Cahalin et al., 2022; Li et al., 2025). Importantly, satellite cell expansion appears to be sensitive to both the metabolic stress and the mechanical signal generated by BFR, suggesting once again that the technique works through hybrid mechanotransduction. The clinical implications of this finding are profound, particularly for populations with compromised regenerative capacity.

Recent work in critically ill survivors illustrates the power of BFR to interrogate satellite cell function in real clinical contexts. Mathur et al. studied intensive care unit survivors with sustained muscle atrophy and applied a short-course, low-intensity BFR regimen to one limb while the contralateral limb served as an internal control (Mathur et al., 2025). The trained vastus lateralis demonstrated significantly higher satellite cell content compared with the untrained contralateral muscle, alongside elevated MuRF1 transcript expression and reduced myostatin expression. Two survivors with particularly low quadriceps mass were able to tolerate the protocol and showed measurable improvements, supporting the feasibility of BFR even in highly deconditioned patients. This case study not only validates a clinically relevant probe for satellite cell function but also illustrates how molecular and clinical findings can be integrated within a single investigation. In healthy ageing populations, satellite cell pool expansion is also a key mechanism by which BFR may attenuate sarcopenia, as Cahalin et al. argued in their systematic review (Cahalin et al., 2022). Recent biological mechanisms reviews have further emphasized that the combination of mTORC1 activation, myostatin suppression, and satellite cell expansion creates a coordinated hypertrophic program that is uniquely accessible at low mechanical loads (Li et al., 2025). The temporal coordination of these events also matters; satellite cell activation typically peaks 24 to 72 hours post-exercise, providing a window in which subsequent training sessions can capitalize on the expanded regenerative pool. This kinetic insight has prompted some authors to recommend BFR session frequencies of three to five times per week to leverage rolling waves of satellite cell activity. As mechanistic understanding deepens, individualized prescription based on satellite cell biology may become increasingly feasible. The trajectory of this research suggests that BFR could become a cornerstone tool in regenerative exercise prescription.

## 4 Endocrine adaptations and anabolic hormonal cascades

### 4.1 Growth hormone and insulin-like growth factor-1 surge

Among the most well-characterized systemic responses to BFR exercise is the rapid and substantial elevation in circulating growth hormone, which can exceed baseline values by an order of magnitude or more in young healthy participants (Takarada et al., 2000; Li et al., 2021). The classical work of Takarada et al. established that BFR resistance exercise produced plasma growth hormone responses up to approximately 290 times resting (pre-exercise) baseline levels, a magnitude measured relative to rest rather than to a non-occluded exercise control, an effect that has since been replicated and refined (Takarada et al., 2000). The proposed mechanism centers on metabolite accumulation, particularly hydrogen ions and lactate, which stimulate group III and IV muscle afferent fibers that in turn drive hypothalamic-pituitary growth hormone release (Suga et al., 2009; Jing et al., 2024). Insulin-like growth factor-1, the principal mediator of growth hormone’s peripheral anabolic effects, also rises modestly following BFR exercise, although the magnitude and consistency of this response are more variable across studies. Li et al. demonstrated a clear dose-response relationship, showing that BFR resistance exercise at 70% of arterial occlusion pressure produced significantly greater post-exercise increases in growth hormone, insulin-like growth factor-1, and testosterone than the same exercise at 40% of arterial occlusion pressure (Li et al., 2021). Such findings indicate that pressure is not merely a safety variable but a hormonal modulator. Importantly, mechanistic studies have shown that local muscle insulin-like growth factor-1 signaling, including the mechano-growth factor isoform, may be more relevant to hypertrophy than systemic levels (Pearson and Hussain, 2015). This local-systemic dichotomy continues to drive nuanced research into the role of the growth hormone/insulin-like growth factor-1 axis in BFR adaptation. The picture that emerges is one of robust acute hormonal activation with complex, partially decoupled, downstream effects.

The functional significance of post-exercise growth hormone elevations has been the subject of ongoing debate, particularly given that the hypertrophic effects of BFR cannot be solely explained by these systemic hormonal pulses. A study by Lopes et al. found that despite eight weeks of low-load BFR or high-load training producing similar increases in muscle cross-sectional area and strength, post-exercise hormonal levels did not correlate with the training-induced changes (Lopes et al., 2022). This finding aligns with broader skepticism toward the so-called hormone hypothesis of muscle hypertrophy and suggests that local muscle-level signaling may dominate over systemic endocrine effects. Nevertheless, growth hormone exerts important roles in connective tissue remodeling, lipolysis, and bone mineralization, all of which may contribute to the broader systemic benefits of BFR training. Acute and chronic BFR resistance exercise has been shown to alter bone metabolic markers, with elevations in bone-specific alkaline phosphatase and reductions in resorption markers reported in young men (Bemben et al., 2022). These observations are particularly relevant for older adults at risk of osteoporotic fracture, in whom anabolic stimuli to bone are difficult to deliver through high-load training. In low-intensity suspension training combined with BFR in young women an uncommon modality in which bodyweight, closed-kinetic-chain suspension exercise is performed under occlusion growth hormone and IGF-1 elevations were associated with measurable improvements in physical fitness over the training period (Sharifi et al., 2024). This pairing is mechanistically notable because it superimposes the metabolic stress of occlusion onto multi-joint stabilizing demands, broadening the contexts in which BFR can be applied. These multifaceted effects suggest that even if growth hormone is not the proximal driver of muscle hypertrophy, its elevation contributes to a broader systemic anabolic milieu. As mechanistic understanding deepens, the challenge will be to translate these endocrine signatures into individualized prescription that maximizes the spectrum of musculoskeletal benefits.

### 4.2 Testosterone, cortisol, and catecholamine dynamics

**Figure 3:**
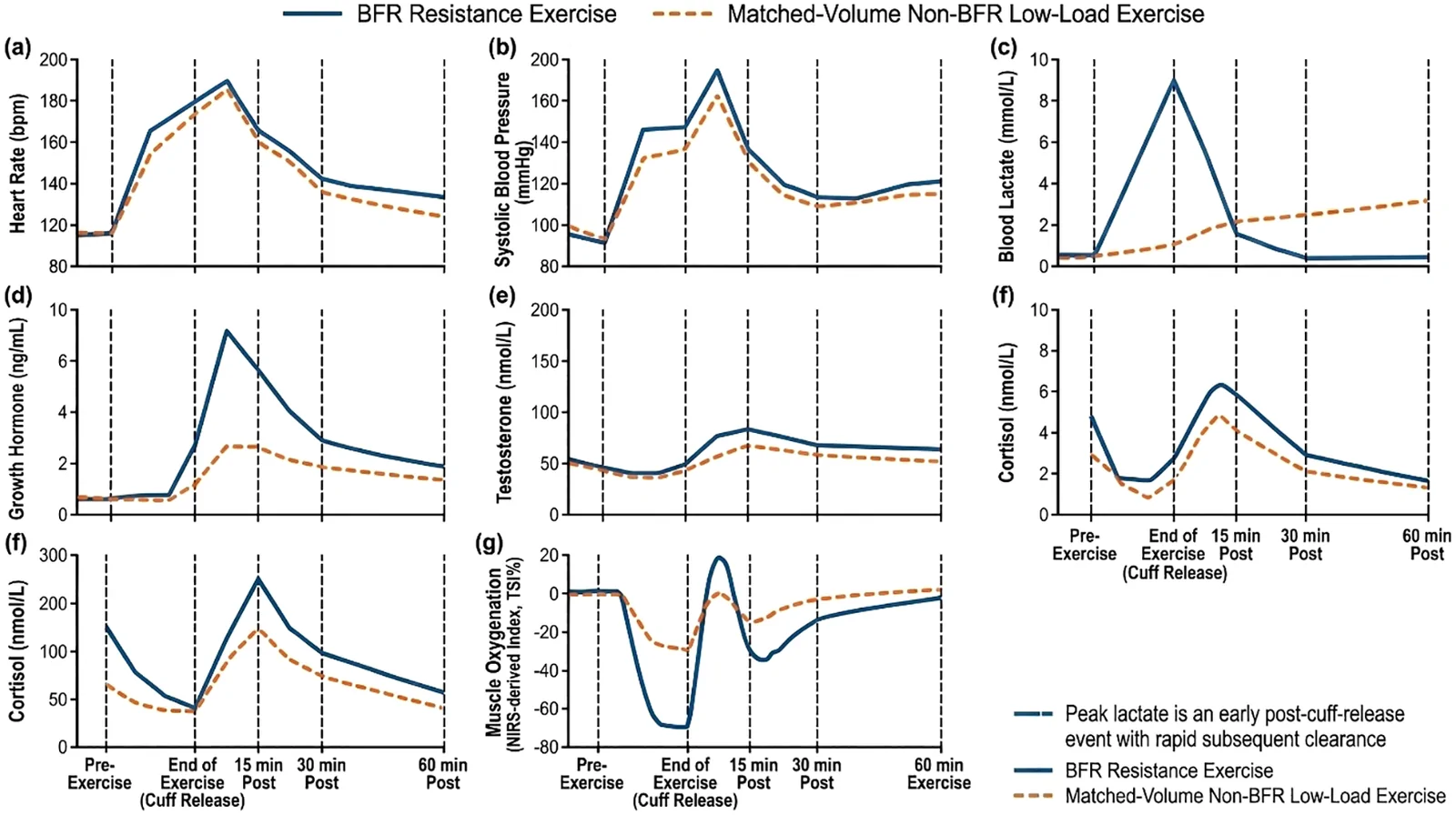
Temporal profile of acute hemodynamic and hormonal responses. This figure is a multi-panel time course showing the acute responses of key physiological variables. Where BFT resistant exercise and matched-volume non-BFR low-load exercise with cuff release and **(a)** heart rate, **(b)** systolic blood pressure, **(c)** blood lactate, **(d)** growth hormone, **(e)** testosterone, **(f)** Cortisol, and **(g)** Muscle oxygenation.

Beyond the growth hormone/insulin-like growth factor-1 axis, BFR exercise affects a broader endocrine ensemble including testosterone, cortisol, and the catecholamines, each of which contributes to the systemic stress and adaptation response. Acute BFR resistance exercise typically produces transient elevations in serum testosterone in young men, although the magnitude is often smaller than that observed with high-load resistance training (Vingren et al., 2010; Li et al., 2021). The interaction between testosterone and BFR-induced metabolic stress remains under investigation, with some evidence suggesting that local androgen receptor sensitivity may rise alongside systemic concentrations. Cortisol responses to BFR are similarly variable; while some protocols produce robust cortisol elevations consistent with metabolic and sympathetic activation, others show minimal change, particularly when total exercise volume is modest (Lopes et al., 2022). Catecholamines, including epinephrine and norepinephrine, rise sharply during BFR exercise as a consequence of metaboreflex activation, sympathetic outflow, and pain perception (Bane et al., 2024; Suggitt et al., 2024). These hormones contribute to acute hemodynamic responses, including heart rate and blood pressure elevations, while also stimulating glycogenolysis and lipolysis in active and remote tissues (**Figure 3**). Notably, the catecholamine surge may also play a role in muscle fiber recruitment, as adrenergic signaling enhances motor unit excitability and modulates fast-twitch fiber activation under fatiguing conditions (Yasuda et al., 2013). The endocrine signature of BFR therefore reflects an integrated acute stress response. This response varies substantially with cuff pressure, exercise volume, and individual physiological background.

Sex- and age-specific differences in endocrine responses to BFR exercise have begun to receive deserved attention, with implications for protocol individualization. Heart rate variability studies have shown that autonomic responses to BFR resistance exercise differ subtly between trained men and women, with women exhibiting smaller post-exercise reductions in vagal tone (Perlet et al., 2026). Such findings raise the possibility that women may experience different sympathoadrenal kinetics following BFR, with potential implications for catecholamine-mediated effects on muscle and cardiovascular tissues. Older adults represent another group whose hormonal milieu is fundamentally different from young controls, with reduced baseline testosterone and growth hormone secretion. In this context, BFR’s ability to elicit even modest acute elevations in anabolic hormones may carry disproportionate clinical importance, as these surges contribute to a more favorable anabolic environment in tissues that are otherwise insulated from anabolic signaling (Cahalin et al., 2022; Li et al., 2025). In an instructive case from the rehabilitation literature, postoperative patients undergoing anterior cruciate ligament reconstruction have been shown to experience hormonal disruptions, including suppressed testosterone and elevated cortisol, that contribute to muscle atrophy. The integration of BFR within these patients’ rehabilitation programs has been associated with improved hormonal balance and functional recovery, as exemplified by the rapid restoration of thigh girth and lean mass in the previously cited fifteen-year-old soccer player (Solie et al., 2023). The downstream effects of catecholamines on the cardiovascular system, however, demand careful attention in clinical contexts, particularly when applying BFR to individuals with hypertension or heart failure. Tailoring protocols to balance anabolic hormonal benefits against sympathoadrenal stress remains an active research priority. As personalized BFR prescription matures, hormonal phenotyping may become a useful adjunct to mechanical and metabolic dosing decisions.

Acute endocrine responses to BFR exercise are variable and protocol-dependent. Some studies report transient post-exercise elevations in growth hormone, testosterone, and cortisol, whereas others report minimal change, and the magnitude depends on exercise volume, load, pressure, and the population studied. Reported acute growth-hormone elevations span a very wide range across protocols; testosterone and cortisol responses are smaller and frequently inconsistent, with several studies showing no significant change. Importantly, these transient hormonal surges have not been reliably shown to drive subsequent hypertrophy, and current evidence favors local mechanical and metabolic signaling over systemic hormonal exposure as the principal driver of BFR adaptation. We therefore present these endocrine responses as variable accompaniments to BFR rather than as established mediators of its effects.

### 4.3 Bone markers and osteogenic hormones

The endocrine response to BFR extends beyond the muscle-centric anabolic axis to include bone-active hormones, a topic of increasing interest given the high prevalence of osteopenia in populations who would benefit most from BFR. Acute BFR resistance exercise has been associated with transient elevations in markers of bone formation, including bone-specific alkaline phosphatase and procollagen type 1 N-terminal propeptide, alongside attenuated resorption markers such as carboxy-terminal telopeptide of type I collagen (Bemben et al., 2022). These shifts are mechanistically attributable to a combination of localized mechanical strain, growth hormone elevation, and possibly to direct effects of metabolite accumulation on osteoblast and osteocyte signaling. Although the chronic effects of BFR on bone mineral density in humans remain less well established, the acute molecular signals are consistent with a pro-osteogenic environment. Osteocalcin, a marker that links bone metabolism with energy regulation, has also been reported to rise following BFR resistance exercise, suggesting integrative effects across multiple endocrine axes. The relevance of these bone-active responses is particularly salient for older adults with osteopenia or osteoporosis, in whom traditional high-impact and high-load exercise carries substantial fracture risk (Cahalin et al., 2022). For these individuals, BFR offers a means of generating bone-relevant mechanical and endocrine signals at low mechanical loads, theoretically reducing the risk of acute injury. However, robust randomized trials evaluating bone mineral density outcomes following long-term BFR training remain relatively scarce. Filling this evidentiary gap will be important for establishing BFR as a recognized intervention for bone health.

The interaction between bone-active hormones and the broader BFR endocrine milieu also deserves consideration in special populations such as postmenopausal women and patients with chronic glucocorticoid exposure. Postmenopausal women experience accelerated bone loss attributable to estrogen decline, and high-load resistance training is often poorly tolerated owing to joint and connective tissue limitations. In this context, low-load BFR resistance exercise has been shown to elicit hormonal and bone marker responses comparable to those observed in younger participants, suggesting that the technique may serve as a viable osteogenic intervention (Bemben et al., 2022). Older patients undergoing rehabilitation following tibial plateau fractures, as recently described by Cao et al., exemplify the practical relevance of these observations; following short-term BFR training, these patients demonstrated improvements in knee function and quality of life that may partially reflect favorable bone-muscle endocrine cross-talk (Cao et al., 2026). The interplay between glucocorticoid signaling and BFR-induced hormonal responses is also important; patients on chronic corticosteroid therapy often suffer from secondary sarcopenia and osteopenia, and emerging evidence suggests that BFR may partially counteract these adverse effects through coordinated muscle and bone hormonal modulation. Although the evidence base in this area is still maturing, the convergence of muscle-centric and bone-centric endocrine signals positions BFR as an integrated musculoskeletal intervention. Future research that explicitly examines the longitudinal endocrine and structural responses of bone to BFR will be valuable. Such studies should ideally employ both serum biomarkers and high-resolution imaging modalities to capture the breadth of the response. As clinical applications expand, multidisciplinary collaboration between endocrinologists, orthopedists, and exercise physiologists will be essential.

## 5 Metabolic stress and bioenergetic reprogramming

### 5.1 Lactate accumulation and acid-base disturbance

Metabolic stress is widely regarded as the central driver of BFR-induced adaptations, and lactate accumulation provides one of its most readily measurable signatures. During BFR resistance exercise, the combination of partial arterial inflow restriction and venous occlusion impairs both oxygen delivery and metabolite washout, creating a localized environment of accelerated glycolysis and rapid lactate production (Suga et al., 2009; Lin et al., 2025). Blood lactate concentrations following BFR resistance exercise can rise to levels approaching those seen following near-maximal high-intensity training, even though the absolute mechanical work performed is far lower. This metabolite accumulation is accompanied by a fall in intracellular pH, which has direct effects on protein kinase activation, calcium handling, and motor unit recruitment patterns. The mechanism by which lactate itself influences anabolic signaling has been the subject of intense investigation, with recent evidence suggesting that lactate may act not merely as a metabolic byproduct but as a signaling molecule modulating the mTORC1 pathway, monocarboxylate transporter expression, and even gene transcription via lactate-mediated lysine lactylation (Pearson and Hussain, 2015; He et al., 2025). Beyond its metabolic role, lactate has been proposed to act as a signaling molecule, for example, as a substrate for histone lactylation and as a ligand at hydroxycarboxylic-acid receptors that could in principle contribute to the adaptive response to BFR. It should be emphasized, however, that this evidence derives largely from cell and animal models, and that a causal role for lactate-mediated signaling or lactylation in human BFR adaptation has not been demonstrated. We therefore present these pathways as plausible but unproven hypotheses that warrant dedicated investigation, rather than as established mechanisms of BFR-induced adaptation. The resulting metabolic and signaling milieu primes muscle fibers for anabolic responses that would otherwise require much higher mechanical loads. This phenomenon has earned BFR the description of a metabolic mimic of high-intensity training, although the parallel is incomplete and the mechanisms only partially overlap. The functional implications of these acid-base shifts extend beyond the trained limb, contributing to systemic ventilatory and circulatory responses. The body’s integrated response to BFR therefore reflects a coordinated metabolic-cardiovascular-endocrine signature.

For clinicians seeking a practical reference, blood lactate after low-load BFR resistance exercise commonly reaches approximately 4–7 mmol·L^-1^, and can exceed this with higher pressures or volumes, broadly comparable to moderate-to-hard conventional exercise. We caution, however, that the relationship between an absolute lactate value and the magnitude of adaptation is not precisely defined; rather than aiming for a fixed number, a perceptible but tolerable metabolic stress consistent with the session goal and the patient’s tolerance is a more defensible practical target, with lactate used as a relative monitoring aid rather than a prescriptive endpoint. The dose-response relationship between cuff pressure, exercise volume, and metabolite accumulation has been increasingly clarified by recent studies that incorporate continuous lactate, pH, and near-infrared spectroscopy monitoring. In a meta-analysis by Lin et al., BFR exercise consistently elicited higher peak lactate concentrations than matched-volume non-occluded exercise across diverse populations and protocols (Lin et al., 2025). Higher cuff pressures produced steeper lactate accumulations and more pronounced acid-base disturbances, suggesting that pressure is a graded modulator of metabolic stress (Li et al., 2021). However, the magnitude of metabolic stress required to maximize anabolic signaling without imposing disproportionate cardiovascular strain remains unsettled. In the case of older adults rehabilitating tibial plateau fractures previously cited, conservative BFR protocols still produced clinically meaningful outcomes despite generating less aggressive lactate spikes (Cao et al., 2026). The temporal kinetics of lactate response to BFR also matter; peak concentrations are typically observed within minutes of cuff release, with rapid clearance occurring during the recovery period. This pattern of accumulation and rapid clearance may itself constitute a useful training stimulus, as it generates repeated waves of metabolite-driven signaling. In low-load resistance exercise performed with BFR, improvements in oxidative capacity and muscular endurance have been reported alongside elevated metabolic stress, suggesting that BFR confers metabolic in addition to anabolic benefits (Davis et al., 2024). The integration of lactate measurement into BFR prescription thus offers a practical biomarker for individualizing dose, particularly in clinical populations whose tolerance for metabolic stress varies widely. As lactate biology continues to evolve from a marker of fatigue to a signaling molecule, our appreciation for its role in BFR adaptation will likely deepen further.

### 5.2 Hypoxia-inducible factor-1α pathway activation

Localized tissue hypoxia is a defining feature of the BFR environment, and the cellular response to this oxygen scarcity is orchestrated largely by hypoxia-inducible factor-1 alpha, a master transcriptional regulator of hypoxic gene expression. Under normoxic conditions, hypoxia-inducible factor-1 alpha is rapidly hydroxylated by prolyl hydroxylase domain proteins, ubiquitinated by the von Hippel-Lindau complex, and degraded within minutes (Larkin et al., 2012; Ferguson et al., 2018). When intracellular oxygen tension falls, as occurs robustly during BFR exercise, this degradation pathway is inhibited, allowing hypoxia-inducible factor-1 alpha to accumulate, dimerize with hypoxia-inducible factor-1 beta, and translocate to the nucleus where it transactivates over one hundred target genes (Ferguson et al., 2018; He et al., 2025). Among the most relevant targets for muscle adaptation are vascular endothelial growth factor, glucose transporter-1, and several glycolytic enzymes, all of which contribute to the metabolic and angiogenic remodeling that follows BFR training. Recent meta-analytic work confirms that BFR exercise elicits significantly greater hypoxia-inducible factor-1 alpha mRNA expression than non-occluded exercise, with resistance modalities producing larger effects than aerobic modalities (Larkin et al., 2012; Cumpston et al., 2022). The pathway’s activation appears to be tightly coupled to the degree of muscle deoxygenation, which is itself a function of cuff pressure, exercise intensity, and capillary density. In addition to its transcriptional roles, hypoxia-inducible factor-1 alpha modulates erythropoiesis and iron handling, with potential implications for whole-body oxygen transport in chronic BFR users. The integrative role of hypoxia-inducible factor-1 alpha thus places it at the intersection of metabolic, vascular, and erythroid adaptation.

The angiogenic consequences of hypoxia-inducible factor-1 alpha activation are particularly relevant to the long-term cardiovascular and muscular adaptations observed after BFR training. Vascular endothelial growth factor and its primary receptor, vascular endothelial growth factor receptor 2, are upregulated at the mRNA level following acute BFR resistance exercise, and chronic training has been associated with increases in capillary-to-fiber ratios in human skeletal muscle (Larkin et al., 2012; Cumpston et al., 2022). These adaptations enhance oxygen and substrate delivery to muscle fibers and may contribute to improvements in both endurance and recovery capacity. Endothelial nitric oxide synthase expression also rises after BFR, supporting improved vasodilatory capacity and shear-stress-mediated remodeling. In a controlled trial described by Cumpston et al., low-load BFR resistance exercise produced angiogenic gene-expression patterns greater than those observed with low-load training without occlusion, and in some respects exceeding even high-load training, although the number of studies is small and the strength of this evidence remains limited (Cumpston et al., 2022). The clinical implications of these findings are substantial, particularly for patients with peripheral artery disease, microvascular dysfunction, or post-traumatic muscle ischemia. The case of older patients recovering from tibial plateau fracture again illustrates this point; restoration of capillary density and microvascular function is essential for full functional recovery, and BFR offers a low-impact means of stimulating these remodeling processes (Cao et al., 2026). The integrated activation of hypoxia-inducible factor-1 alpha across muscle and vascular tissues may also explain why BFR training is associated with improvements in markers of metabolic health, including insulin sensitivity. Continued investigation into the pleiotropic effects of this pathway will likely reveal additional therapeutic implications. Future trials should incorporate biomarkers of vascular remodeling alongside traditional muscle outcomes.

### 5.3 Mitochondrial function and oxidative capacity

Although BFR is most often associated with hypertrophic adaptations, evidence increasingly indicates that it also enhances mitochondrial function and oxidative capacity through pathways involving AMP-activated protein kinase, peroxisome proliferator-activated receptor gamma coactivator 1-alpha, and reactive oxygen species signaling. AMP-activated protein kinase is activated under conditions of energy depletion, and its activation by BFR exercise reflects the exaggerated AMP-to-ATP ratio that develops in restricted muscle (Petrick et al., 2023; Chen et al., 2025). Peroxisome proliferator-activated receptor gamma coactivator 1-alpha, the master regulator of mitochondrial biogenesis, is upregulated in response to AMP-activated protein kinase activation and to elevated cytosolic calcium concentrations, both of which are amplified by BFR exercise. The downstream effect is increased mitochondrial content and enhanced oxidative phosphorylation capacity, particularly when BFR is combined with aerobic exercise modalities. Davis et al. conducted a pilot investigation comparing low-load resistance exercise with and without BFR for effects on muscle strength, endurance, and oxidative capacity, finding that BFR amplified some adaptations but produced complex patterns that varied by outcome (Davis et al., 2024). These findings reinforce the view that mitochondrial responses to BFR are sensitive to specific protocol parameters. Notably, the localized hypoxia of BFR may transiently limit reactive oxygen species production at the mitochondrial level, potentially altering the redox-mediated signaling that traditionally accompanies high-intensity exercise (Petrick et al., 2023). The mitochondrial response to BFR therefore reflects a unique integration of energetic, redox, and transcriptional inputs.

The functional implications of BFR-induced mitochondrial adaptation extend beyond endurance performance to include broader metabolic health benefits. Improvements in mitochondrial oxidative capacity are linked to enhanced insulin sensitivity, reduced inflammation, and improved fatty acid oxidation, all of which are particularly relevant for populations with metabolic disease (Chen et al., 2025; Li et al., 2025). In aerobic BFR training, where treadmill walking or cycling is performed under cuff inflation, recent meta-analytic work has demonstrated greater improvements in maximal oxygen uptake compared with matched aerobic training without occlusion (Chen et al., 2025). These adaptations occur even at relatively low aerobic loads, making BFR aerobic protocols attractive for populations who cannot tolerate high-intensity aerobic exercise. The case of patients with chronic obstructive pulmonary disease, recently reviewed by Alves et al., exemplifies this clinical promise; BFR has been shown to improve exercise capacity and muscle strength in these patients, with potential mechanisms including mitochondrial biogenesis and improved peripheral muscle function (Alves et al., 2026). Importantly, BFR aerobic exercise may also enhance the response to traditional aerobic training when used as an adjunct, periodized in alternation with non-occluded sessions. The integration of BFR into combined training programs has been shown to elicit cross-adaptive benefits, with metabolic stress phases supporting type I fiber adaptation and high-load phases supporting type II fiber recruitment (He et al., 2025). The bioenergetic plasticity engaged by BFR is therefore a versatile tool for shaping adaptation across populations and goals. As mitochondrial science continues to evolve, BFR’s role in metabolic medicine will likely expand correspondingly. Future investigations integrating mitochondrial functional assays with clinical outcomes will be particularly valuable.

## 6 Cellular stress responses and redox signaling

### 6.1 Reactive oxygen species and antioxidant defense

Reactive oxygen species, once viewed exclusively as damaging byproducts, are now recognized as essential signaling molecules in skeletal muscle adaptation, and BFR exercise provides a unique context in which to examine their role. The cyclic ischemia-reperfusion pattern inherent to BFR creates conditions under which reactive oxygen species production rises during the contraction phase and increases further upon cuff release as oxygen returns to previously hypoxic tissue (Ferlito et al., 2023; Ferreira et al., 2024). This pattern resembles the ischemia-reperfusion injury seen in pathological contexts but, when controlled and dosed appropriately, may instead trigger hormetic adaptations that strengthen antioxidant defenses and amplify anabolic signaling. A meta-analysis by Ferlito et al. found that acute low-load BFR resistance exercise produced reactive oxygen species responses similar to or greater than those observed with non-occluded equivalent exercise, although chronic adaptation to repeated BFR was associated with enhanced antioxidant capacity (Ferlito et al., 2023). Markers of oxidative stress including lipid peroxidation, protein carbonyls, and reduced glutathione depletion follow predictable patterns that depend on whether BFR is partial or total. Total occlusion, which produces complete arterial blockade, generates substantially greater oxidative stress than partial occlusion at typical clinical pressures (Garten et al., 2021). This distinction has important implications for both safety and efficacy, as excessive oxidative stress may promote tissue damage rather than adaptation. The redox biology of BFR is therefore a critical determinant of whether the technique is constructive or potentially harmful.

The interplay between reactive oxygen species, mitochondrial signaling, and downstream anabolic pathways is increasingly understood to be a key element of BFR adaptation, and contemporary research has begun to reveal a biphasic temporal profile. Albumin Cys34 oxidation, used as a sensitive marker of oxidative stress, has been shown to follow a biphasic pattern after low-load BFR resistance exercise, with an early peak immediately post-exercise and a secondary rise several hours later (Ferreira et al., 2024). This biphasic profile reflects an initial burst of contraction-driven reactive oxygen species production followed by a delayed wave of mitochondrial and inflammatory reactive oxygen species generation. Such kinetics may explain why some studies report transient oxidative stress while others find sustained elevations in markers of redox imbalance. Importantly, antioxidant supplementation studies have suggested that blunting reactive oxygen species production with high-dose antioxidants may attenuate some of the adaptive benefits of BFR training, supporting the view that controlled oxidative stress is integral to the training response (Christiansen et al., 2018). The case of post-surgical patients again illustrates these principles; after anterior cruciate ligament reconstruction, patients exhibit elevated baseline oxidative stress that may be modulated by structured BFR rehabilitation, supporting both muscle regeneration and microvascular adaptation (Solie et al., 2023; Butt and Ahmed, 2024). The redox response to BFR is also influenced by training status, age, and underlying disease, with older adults and chronically ill patients exhibiting blunted antioxidant capacity that may require more conservative dosing. Continued mechanistic work into the redox biology of BFR will be critical for defining safe and effective protocols. As individualized prescription matures, redox biomarkers may become useful adjuncts to traditional cardiovascular and biochemical monitoring.

### 6.2 Heat shock protein and AMPK activation

In addition to reactive oxygen species signaling, BFR exercise activates classical cellular stress response pathways including heat shock protein expression and AMP-activated protein kinase signaling. Heat shock proteins, particularly heat shock protein 70 and the small heat shock protein alpha-B-crystallin, are induced in response to thermal, mechanical, and metabolic stresses, and their expression has been documented after BFR resistance exercise (Cumming et al., 2014). These proteins serve chaperone functions, supporting protein folding and protecting against aggregation under stress conditions, and their induction is associated with improved muscle protein quality control and resilience to subsequent stressors. The activation of AMP-activated protein kinase under BFR conditions reflects the dramatic shift in cellular energy state induced by combined contraction and ischemia (Petrick et al., 2023; He et al., 2025). AMP-activated protein kinase activation has historically been viewed as antagonistic to mTORC1, owing to its inhibitory effects on protein synthesis when activated chronically; however, recent evidence suggests that transient AMP-activated protein kinase activation following BFR exercise may coexist with mTORC1 activation and contribute to mitochondrial biogenesis without necessarily inhibiting hypertrophy (Furrer and Handschin, 2024); BFR-specific work supports AMPK and PGC-1α signaling after blood flow–restricted exercise (Christiansen et al., 2018). We note that the apparent co-existence of AMPK and mTORC1 activation remains partially contested, as canonical models position AMPK as an mTORC1 inhibitor. The dual activation of these pathways may explain why BFR can simultaneously improve hypertrophic and oxidative adaptations, a combination that is rarely achieved with other training modalities. The integration of these stress responses with metabolic and growth signaling makes BFR a uniquely versatile stimulus. The cellular stress signature of BFR is therefore broader than that of either traditional resistance or aerobic training alone.

**Figure 4:**
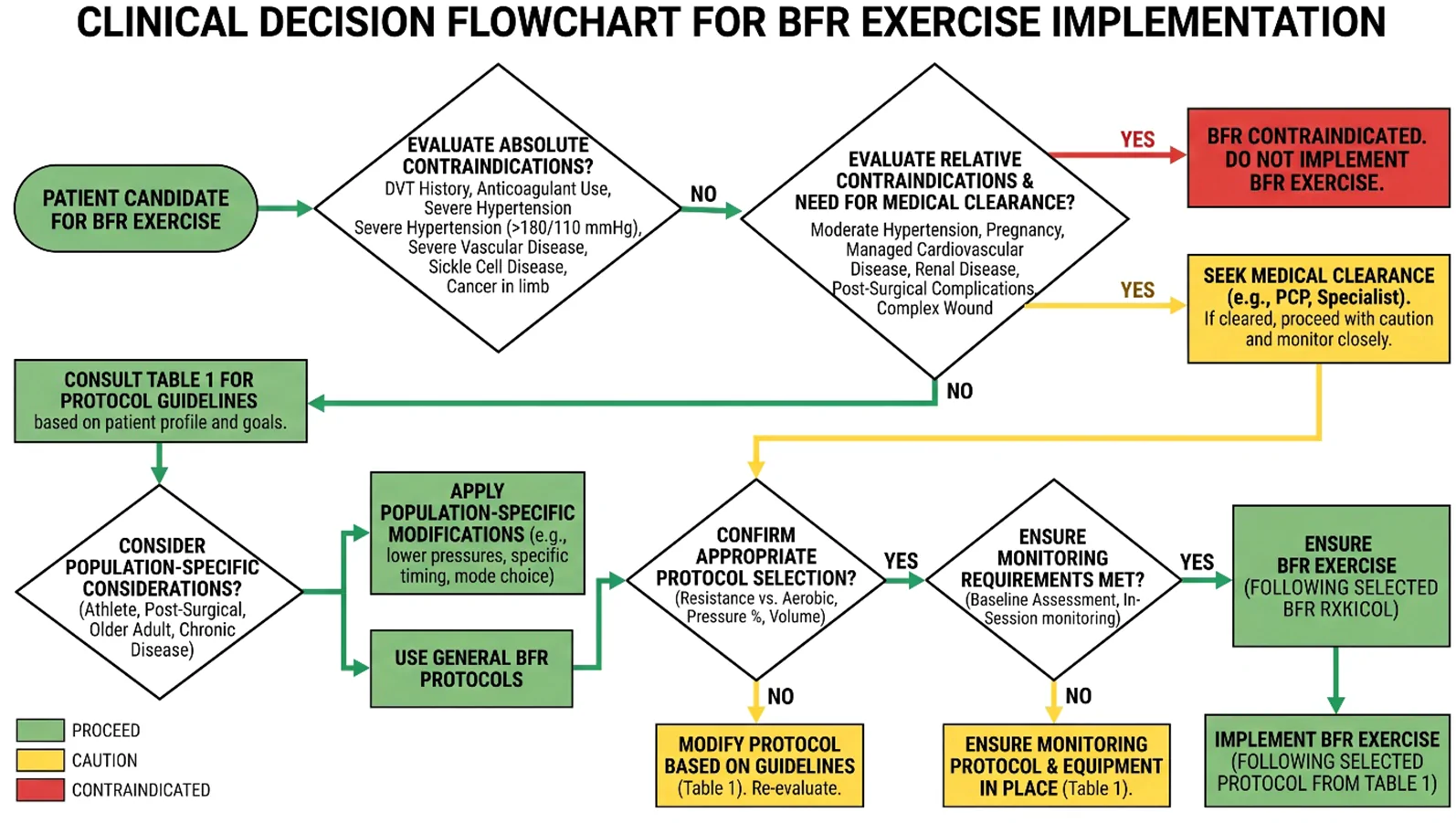
Clinical decision tree for BFR implementation. This flowchart guides clinicians through the decision to implement BFR exercise.

The relevance of heat shock protein and AMP-activated protein kinase activation extends beyond acute signaling to chronic adaptations that may protect skeletal muscle from injury, atrophy, and disease. Repeated BFR exposure has been associated with elevated baseline heat shock protein expression, which may confer protection against subsequent ischemic, oxidative, or inflammatory stresses. This adaptive resilience is particularly relevant in clinical contexts where muscle is repeatedly exposed to stress, including chronic disease, aging, and post-surgical recovery (Cumming et al., 2014; Mathur et al., 2025). AMP-activated protein kinase activation also has implications for whole-body energy metabolism, including improvements in insulin sensitivity, fatty acid oxidation, and glucose uptake. In the case of older adults with sarcopenia, the combination of heat shock protein induction, AMP-activated protein kinase activation, and reactive oxygen species-mediated signaling provides a multifaceted molecular foundation for the clinical benefits observed in this population (Cahalin et al., 2022; Li et al., 2025). The case of critically ill survivors described by Mathur et al. again provides a vivid illustration; in patients with markedly compromised cellular stress response capacity, even brief BFR exposure produced measurable molecular shifts including changes in MuRF1 and myostatin transcript expression (Mathur et al., 2025). These transcript-level changes likely reflect the integrated activation of stress response pathways that include but are not limited to heat shock proteins and AMP-activated protein kinase. As mechanistic understanding deepens, the molecular signature of BFR adaptation is increasingly viewed as a coordinated stress response rather than a single pathway activation. This integrated perspective is reshaping how researchers and clinicians conceptualize the technique (**Figure 4**). Future work that maps the temporal coordination of stress response pathways will further clarify the molecular logic of BFR adaptation.

### 6.3 Cellular swelling and mechanotransduction

Cellular swelling, induced by the combination of metabolite accumulation and venous congestion, has been proposed as a distinct mechanism by which BFR exercise drives anabolic signaling. Loenneke et al. (2012) hypothesized that the anabolic benefits of venous blood flow restriction may be induced by muscle cell swelling. This proposal remains a hypothesis-generating idea rather than an experimental demonstration: direct experimental confirmation of a swelling-driven anabolic mechanism in humans remains limited, and the concept should be read as plausible but unproven (Loenneke et al., 2012; He et al., 2025). This cell swelling has been hypothesized to act as a mechanical signal that activates integrins, focal adhesion kinases, and downstream pathways converging on protein synthesis and inhibition of proteolysis. While direct experimental confirmation of cell swelling as an independent anabolic stimulus in human BFR exercise remains limited, the parallel evidence from cell culture studies and pharmacological models is suggestive. Mechanotransduction more broadly is engaged through several channels including stretch-activated ion channels, costameric protein complexes, and titin-based signaling, all of which may be amplified under the unique mechanical conditions of BFR (Pearson and Hussain, 2015). The integration of mechanical, osmotic, and metabolic signals at the muscle cell membrane represents a frontier of BFR research. Increasing evidence suggests that these signals converge on common transcriptional programs, including those governing structural and contractile protein synthesis. The convergence of pathways may explain the reproducibility of hypertrophic outcomes across diverse BFR protocols. The mechanotransductive logic of BFR thus provides a unifying framework for many of its observed effects.

**Figure 5:**
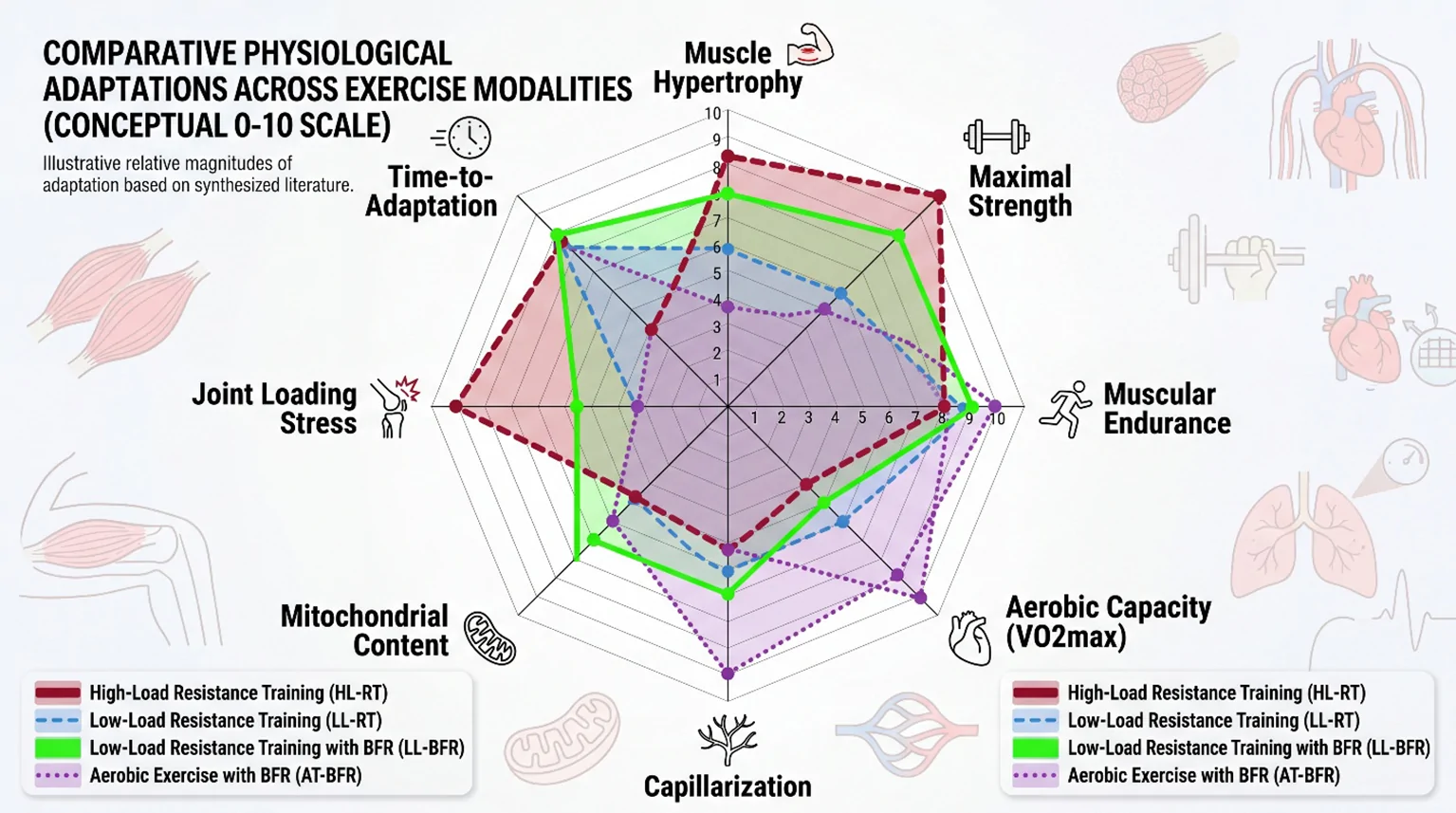
Comparative adaptation profiles across training modalities. This radar/spider chart compares the relative magnitudes of adaptation produced by four modalities: high-load resistance training (HL-RT), low-load resistance training without BFR (LL-RT), low-load resistance training with BFR (LL-BFR), and aerobic exercise with BFR (AT-BFR).

The clinical relevance of cell swelling and mechanotransduction is particularly evident in populations with compromised mechanical signaling, including patients with sarcopenia, post-surgical atrophy, and disuse-related muscle loss. In these contexts, traditional high-load resistance training generates the mechanical signals necessary for adaptation but may be poorly tolerated owing to joint, tendon, or systemic limitations (**Figure 5**). BFR offers an alternative pathway by amplifying mechanical signaling through osmotic and metabolic mechanisms, even when external loads are modest. The case of the fifteen-year-old soccer player rehabilitating from anterior cruciate ligament reconstruction provides an illustrative example; despite weeks of compromised loading, his integration of practical BFR allowed for sufficient mechanotransductive stimulation to drive measurable hypertrophy in the surgical limb (Solie et al., 2023). In older adults with sarcopenia, similar principles apply, with the additional benefit that BFR-induced cell swelling may partially compensate for age-related declines in mechanical sensitivity (Cahalin et al., 2022; Li et al., 2025). The mechanism is also relevant in athletic populations, where BFR can supplement traditional training to drive additional adaptation without further accumulating mechanical stress on already heavily loaded joints (Su et al., 2025). The integration of mechanotransductive insights with metabolic and endocrine considerations therefore positions BFR as a uniquely flexible tool for adaptive prescription. As wearable sensor technology advances, real-time monitoring of muscle thickness, oxygenation, and electromyographic activity may allow clinicians to tune BFR protocols based on integrated mechanotransductive feedback. Such precision-based applications represent the next horizon of BFR research and clinical practice. Continued investment in this area will be essential to fully realize the technique’s potential.

## 7 Cardiovascular and hemodynamic adaptations

### 7.1 Acute hemodynamic responses

The acute cardiovascular response to BFR exercise is one of the most clinically important and most widely studied aspects of the technique, and it reflects an integrated activation of the exercise pressor reflex, central command, and metaboreflex pathways. During BFR resistance exercise, heart rate, systolic blood pressure, and rate-pressure product all rise to magnitudes that often exceed those observed during equivalent non-occluded low-load exercise, although they generally remain below those of high-load training (Lin et al., 2025; Suggitt et al., 2024). Cardiac output increases owing to elevated heart rate, while stroke volume responses are variable and may be slightly attenuated by reduced venous return from the working limb. Systemic vascular resistance rises sharply, reflecting both the cuff-induced peripheral resistance and the centrally mediated sympathetic vasoconstriction. The metaboreflex, activated by the accumulation of muscle metabolites, contributes substantially to these responses and is particularly relevant in patients with cardiovascular disease who exhibit exaggerated reflex activity (Suggitt et al., 2024; Cristina-Oliveira et al., 2020). Recent meta-analytic work by Lin et al. confirmed that BFR elevates heart rate and lactate while exhibiting variable effects on oxygen saturation and stroke volume (Lin et al., 2025). The integrated hemodynamic response to BFR is therefore a coordinated activation of cardiac, vascular, and reflex systems. Importantly, these responses are largely transient and resolve within minutes of cuff release, although recovery kinetics differ across populations and protocols. With respect to arrhythmia specifically, neither the large Japanese KAATSU surveillance series nor controlled trials have demonstrated an increased incidence of clinically significant arrhythmia following BFR exercise in appropriately screened individuals (Nakajima et al., 2006; Suggitt et al., 2024). Isolated case reports exist, and caution remains warranted in people with known arrhythmia or structural heart disease, but a causal increase in arrhythmia attributable to BFR has not been established.

Despite the generally favorable safety profile of BFR exercise in healthy adults, important concerns persist regarding its application to populations with cardiovascular disease, hypertension, or autonomic dysfunction. Suggitt et al. recently emphasized that pre-exercise and post-exercise hemodynamic measurements are inadequate proxies for the cardiovascular responses occurring during BFR exercise itself, and that exaggerated metaboreflex activation in cardiovascular patients warrants particular caution (Suggitt et al., 2024). Heart rate variability following BFR resistance exercise reflects altered autonomic balance, with attenuated post-exercise vagal recovery in some studies; recent work by Perlet et al. compared men and women and reported sex-specific differences in autonomic responses, suggesting that risk stratification may need to be sex-aware (Perlet et al., 2026). In older adults, where autonomic flexibility is generally reduced, network meta-analyses have indicated that low-pressure, low-frequency BFR protocols achieve favorable balance between strength gains and cardiovascular safety (Ren et al., 2025). The case of older patients recovering from tibial plateau fracture again illustrates this principle; in their cohort, conservatively dosed BFR produced clinically meaningful improvements without notable cardiovascular adverse events (Cao et al., 2026). Conversely, in patients with severe hypertension, atrial fibrillation, or congestive heart failure, even low-pressure protocols may produce hemodynamic responses that exceed safe thresholds and require careful monitoring. The integration of point-of-care hemodynamic monitoring, including blood pressure cuffs and pulse oximetry, is increasingly common in clinical BFR practice and supports individualized dosing decisions. As BFR expands into broader clinical settings, formal contraindication lists and risk stratification tools will be essential to ensure safe deployment. Continued research into the determinants of acute hemodynamic responses will help refine these tools.

### 7.2 Vascular endothelial function and arterial compliance

The chronic cardiovascular adaptations to BFR training extend beyond hemodynamic tolerance to include measurable improvements in vascular endothelial function and arterial compliance (**Table 3**). Endothelial function, typically assessed by flow-mediated dilation of the brachial artery, has been shown to improve following BFR resistance training in both healthy adults and clinical populations (Manini et al., 2011; Yasuda et al., 2013). The mechanism centers on repeated bouts of shear stress and metabolite-induced vasodilation, both of which stimulate endothelial nitric oxide synthase activity and improve nitric oxide bioavailability. Arterial compliance, a measure of how readily the arterial tree expands and recoils with each cardiac cycle, is similarly modifiable and has been shown to improve after several weeks of BFR resistance training (Yasuda et al., 2013). The improvements in endothelial function and arterial compliance have implications not only for cardiovascular health but also for muscle perfusion, exercise tolerance, and recovery from injury. Importantly, concerns that BFR might increase arterial stiffness through repeated mechanical compression have largely been allayed by longitudinal studies showing no detrimental effect on central arterial stiffness; Yasuda et al. reported no significant change in central arterial stiffness following blood flow–restricted low-intensity resistance training in older adults, which is best described as a neutral rather than a clearly favorable effect (Yasuda et al., 2013). However, vigilance is warranted in patients with pre-existing endothelial dysfunction or atherosclerosis, where the chronic effects of BFR remain less well characterized. The vascular adaptations to BFR therefore reflect the technique’s potential as a low-load alternative for cardiovascular training. As clinical applications expand, vascular outcome measurement will become an increasingly important component of BFR research.

**Table 3:** Acute and chronic cardiovascular responses to BFR exercise.

Parameter	Acute response	Chronic adaptation	Key source(s)
Heart Rate	↑↑ (greater than non-BFR LL)	↓ resting HR; improved HRV	Lin et al. (2025); Suggitt et al. (2024)
Systolic Blood Pressure	↑↑ (between LL & HL)	↓ or unchanged in healthy adults	Lin et al. (2025); Suggitt et al. (2024)
Cardiac Output	↑ (driven by HR rise)	Improved efficiency at sub-max work	Lin et al. (2025)
Vascular Resistance	↑↑ (cuff + sympathetic)	↓ resting; improved compliance	Suggitt et al. (2024)
Endothelial Function (FMD)	Transient post-occlusion FMD reduction (reactive; measured after cuff release)	Improved flow-mediated dilation after training	Yasuda et al. (2013); Cumpston et al. (2022)
Capillary Density	No acute change	↑ capillary-to-fiber ratio	Cumpston et al. (2022)
VEGF/HIF-1α mRNA	↑↑ within hours post-exercise	Enhanced angiogenic transcriptome	Cumpston et al. (2022)

↑ = increase, ↓ = decrease, double arrows indicate larger magnitude. LL, low-load; HL, high-load; HRV, heart rate variability; FMD, flow-mediated dilation. The endothelial-function row now distinguishes the acute, reactive post-occlusion FMD reduction (measured after cuff release, not during exercise) from the chronic improvement after training.

The angiogenic component of vascular adaptation, addressed in part within the metabolic stress section, reinforces the broader cardiovascular benefits of BFR training. Stimulation of vascular endothelial growth factor and its receptors leads to capillary sprouting and remodeling within trained muscle, increasing the capillary-to-fiber ratio and supporting improved oxygen and substrate delivery (Larkin et al., 2012; Cumpston et al., 2022). Endothelial nitric oxide synthase upregulation contributes both to angiogenesis and to ongoing vasodilatory tone, providing complementary structural and functional improvements. The case of patients with peripheral artery disease, while not yet extensively studied with BFR, represents a logical next frontier; these patients exhibit profound microvascular dysfunction and would theoretically benefit from BFR-induced angiogenic remodeling. In chronic obstructive pulmonary disease, recent meta-analytic work has shown that BFR training improves exercise capacity and muscle strength, and the underlying vascular adaptations likely contribute to these improvements alongside mitochondrial and muscle-specific changes (Alves et al., 2026). The integration of acute hemodynamic responses with chronic vascular adaptations creates a comprehensive picture of cardiovascular plasticity in response to BFR training. Importantly, vascular adaptations may also confer cross-limb and systemic benefits, with some evidence suggesting that BFR training of one limb produces vascular changes in the contralateral untrained limb. This systemic vascular response further supports the view that BFR has effects extending well beyond the trained muscle. As biomarkers of vascular health continue to be refined, BFR’s role in cardiovascular medicine will be increasingly clarified. The technique’s potential as a vascular intervention for populations unable to tolerate conventional cardiovascular training is particularly promising.

### 7.3 Angiogenic gene expression and capillarization

Capillary remodeling is one of the most clinically meaningful chronic adaptations to BFR training, and it depends on the coordinated activation of multiple angiogenic genes and pathways. Vascular endothelial growth factor, the principal driver of angiogenesis, is upregulated at the mRNA level following acute BFR resistance exercise, with effect sizes typically larger than those observed with non-occluded equivalent exercise (Larkin et al., 2012; Cumpston et al., 2022). Vascular endothelial growth factor receptor 2, hypoxia-inducible factor-1 alpha, peroxisome proliferator-activated receptor gamma coactivator 1-alpha, and endothelial nitric oxide synthase are all elevated, creating a transcriptional environment conducive to capillary sprouting and stabilization. Histological studies confirm that chronic BFR resistance training increases capillary density and capillary-to-fiber ratio in human skeletal muscle, although the magnitude of these effects varies with population and protocol. The angiogenic response to BFR resistance exercise appears to exceed that observed with equivalent aerobic exercise alone, possibly reflecting the more pronounced hypoxia and metabolite accumulation generated by occluded resistance exercise (Cumpston et al., 2022). Importantly, when BFR is combined with whole-body vibration or with periodized high-load resistance training, the capillary-to-fiber ratio increases beyond what is achievable with either modality alone. The synergy of BFR with other modalities thus offers a tool for maximizing vascular adaptation. The clinical implications of these findings extend across rehabilitation, geriatric medicine, and chronic disease management.

The functional consequences of BFR-induced angiogenesis include improved oxygen delivery, accelerated metabolite clearance, enhanced recovery between exercise bouts, and improved insulin sensitivity. These benefits are particularly relevant for patients with diabetes, peripheral artery disease, and chronic kidney disease, all of whom exhibit impaired microvascular function. Although direct evidence in these patient populations is still emerging, the mechanistic foundation provided by BFR’s angiogenic signature is compelling. The case of older adults rehabilitating from tibial plateau fractures illustrates how angiogenic adaptations may underlie the broader functional improvements observed; restoration of capillary density supports tissue oxygenation, fluid clearance, and waste removal, all of which contribute to reduced edema and improved knee function (Cao et al., 2026). In athletes, the angiogenic benefits of BFR can support enhanced recovery and tolerance for high training volumes, although the clinical relevance of capillary changes in this population is more incremental (Su et al., 2025). The combination of angiogenesis with mitochondrial biogenesis is particularly powerful, as it allows trained muscle to extract more oxygen and use it more efficiently. This combination is rarely achieved with low-load training alone and represents one of the most distinctive features of BFR adaptation. As longitudinal trials continue to refine the time course of capillary remodeling, optimal training duration and frequency for vascular outcomes will become clearer. The integration of vascular outcomes into clinical BFR research is essential for fully appreciating the technique’s systemic benefits.

## 8 Inflammatory and immunomodulatory responses

### 8.1 Acute cytokine and leukocyte mobilization

The inflammatory and immune responses to BFR exercise have only recently been systematically characterized, and emerging evidence indicates that BFR elicits acute responses comparable to those of high-load resistance training despite its low mechanical stimulus. A 2025 systematic review by Barawi et al. evaluated the inflammatory parameters following BFR resistance training across multiple studies, reporting that, across heterogeneous studies and with high inter-study variability, the peak reported increases reached neutrophils +40%, interleukin-6 + 340%, CD4-positive T cells +25%, and CD8-positive T cells +39% (Barawi et al., 2025). These figures represent the upper bound of reported responses rather than characteristic or expected effects, and should be interpreted in light of substantial differences in cuff pressure, exercise volume, and post-exercise sampling time across the studies. These findings suggest that BFR induces a transient leukocytosis, lymphocytosis, and increased macrophage activity, comparable in magnitude to high-load resistance training. The mechanism appears to be primarily metabolic stress-driven, as the localized accumulation of metabolites, hypoxia, and reactive oxygen species generates damage-associated molecular patterns that activate innate immune signaling. Interleukin-6, often elevated dramatically following BFR exercise, plays a dual role as a myokine that supports muscle adaptation and as an inflammatory cytokine that, if sustained, can contribute to systemic inflammation (Barawi et al., 2025; He et al., 2025). The rapid clearance of interleukin-6 following acute BFR exercise is therefore considered favorable, supporting adaptive signaling without prolonged inflammatory exposure. Other cytokines including tumor necrosis factor-alpha and interleukin-1 beta have been examined less consistently but appear to follow similar transient patterns. The acute immune response to BFR thus reflects a coordinated and largely physiological activation of innate and adaptive systems.

The clinical significance of acute immune responses to BFR exercise extends beyond muscle adaptation to encompass broader systemic effects on immunosurveillance and inflammation. Transient leukocyte mobilization following BFR exercise is consistent with the demargination of cells from the vascular endothelium and from peripheral lymphoid tissues, a phenomenon also observed with traditional exercise. Variability in these responses across studies is attributable in part to differences in cuff pressure, exercise volume, and the timing of post-exercise blood sampling. The variability also underscores the importance of standardized reporting in BFR research, particularly with respect to the precise specifications of the occlusion stimulus (Barawi et al., 2025). The case of critically ill survivors described by Mathur et al. is again instructive; in patients whose immune systems have been chronically dysregulated by intensive care unit-acquired weakness, BFR exercise must be carefully dosed to avoid exacerbating systemic inflammation while still capturing its anabolic benefits (Mathur et al., 2025). Similarly, in patients with autoimmune conditions or chronic inflammatory disease, the risk-benefit analysis of BFR exercise requires careful attention to baseline inflammatory status. Encouragingly, the available evidence suggests that BFR does not produce sustained inflammatory elevations beyond those observed with traditional exercise, and may even support immune homeostasis through the resolution-promoting effects of repeated transient inflammatory pulses. Continued investigation into the resolution phase of BFR-induced inflammation will be valuable for refining clinical applications (**Table 4**). As immune profiling technologies advance, more granular characterization of BFR-induced immune responses will likely emerge.

**Table 4:** Clinical applications of BFR exercise: evidence summary.

Population	Primary outcomes	Key mechanisms	Key reference(s)	Evidence level
ACL reconstruction	↑ thigh CSA, knee-extensor strength	mTORC1, satellite cells, ↓ atrophy genes	Butt and Ahmed (2024); Solie et al. (2023)	SR/MA (Level I); case illustrative
Sarcopenia/older adults	↑ grip strength, gait speed, 1RM	Anabolic-resistance offset; satellite cells	Cahalin et al. (2022); Li et al. (2025); Ren et al. (2025)	SR/MA (Level I)
Tibial plateau fracture (older)	↑ knee function, quality of life	Hypertrophy, angiogenesis, pain modulation	Cao et al. (2026)	Single cohort (Level III)
ICU-acquired weakness	↑ satellite-cell content, ↓ myostatin	Satellite-cell function probe	Mathur et al. (2025)	Mechanistic pilot (Level IV)
COPD	↑ 6MWT, muscle strength, ↓ fatigue	Mitochondrial biogenesis, angiogenesis	Alves et al. (2026)	SR/MA (Level I)
Knee osteoarthritis	↓ pain, ↑ function, ↑ quad strength	Hypoalgesia, anti-inflammation, hypertrophy	Xie et al. (2025)	Mini-review/SR (Level I–II)
Chronic low back pain	↑ multifidus thickness, muscle activity	Hypertrophy, neuromuscular activation	Werasirirat et al. (2026)	RCT (Level II)
Hemiplegia/post-stroke	↑ muscle mass, walking capacity	Hypertrophy, neuromuscular activation	Feng et al. (2025)	RCT (Level II)
Athletes (performance)	↑ jump, sprint, 1RM	Type II fiber recruitment, mTOR	Su et al. (2025); He et al. (2025)	SR/MA (Level I)

CSA, cross-sectional area; 1RM, one-repetition maximum; 6MWT, six-minute walk test; COPD, chronic obstructive pulmonary disease; ICU, intensive care unit; SR/MA, systematic review/meta-analysis. Evidence Level reflects the strongest available design for that application (Level I = SR/MA of RCTs; II = RCT; III = controlled cohort; IV = case/mechanistic pilot).

### 8.2 Chronic immune adaptation and resolution

Beyond the acute responses, chronic BFR training may shape the immune system through repeated cycles of activation and resolution, although evidence in this area remains less mature than the acute literature. Studies of long-term BFR training in healthy adults have generally shown neutral or favorable effects on baseline inflammatory markers including C-reactive protein, interleukin-6, and tumor necrosis factor-alpha, suggesting that the technique does not promote chronic low-grade inflammation. Some investigations have reported reductions in baseline pro-inflammatory cytokines following BFR training programs, paralleling the anti-inflammatory effects of traditional aerobic and resistance training. The mechanism likely involves coordinated adaptations in the muscle-immune axis, including changes in macrophage polarization, regulatory T cell function, and myokine secretion patterns (Barawi et al., 2025; He et al., 2025). Importantly, BFR may also enhance the resolution of inflammation, a process distinct from simple suppression that involves the active production of resolving lipid mediators and the recruitment of regulatory immune cells. This resolution-promoting effect would be particularly valuable in clinical contexts where chronic low-grade inflammation contributes to disease progression. The integration of immune adaptation into BFR’s broader systemic profile reinforces the technique’s potential as a comprehensive systemic intervention. As mechanistic understanding of resolution biology deepens, BFR research is likely to capitalize on these insights. It is worth noting that this proposed mechanism is speculative and is offered as a hypothesis to guide future work rather than as an established pathway. The supporting evidence is indirect, and the concept should be tested directly, ideally in controlled human BFR studies with appropriate molecular endpoints before it is incorporated into mechanistic models of BFR adaptation.

**Figure 6:**
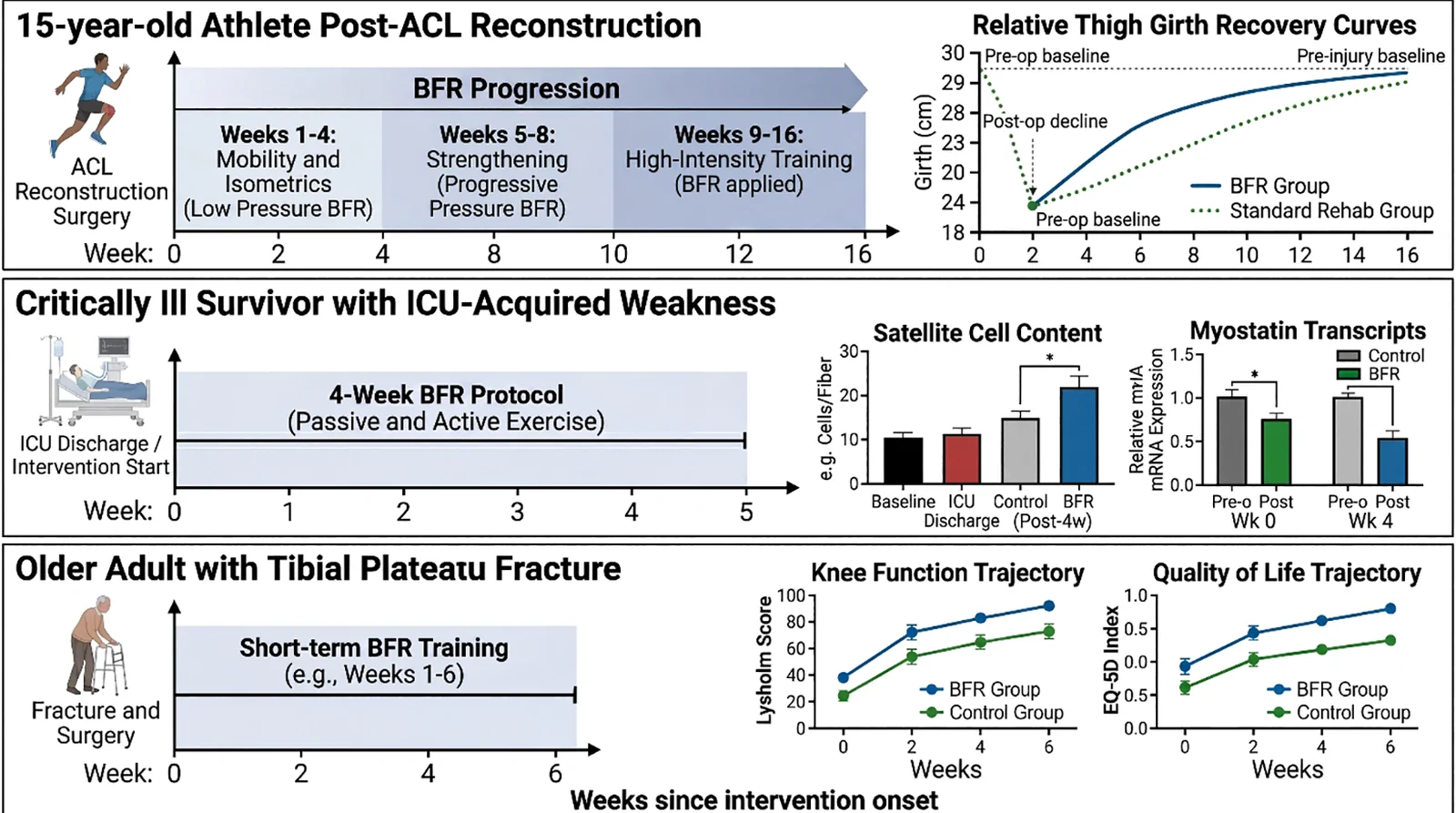
Case study integration: BFR across the rehabilitation continuum which illustrate three contrasting cases discussed throughout the review on a single timeline figure.

The interaction between immune adaptation and other systemic responses to BFR is increasingly recognized as a key determinant of training outcomes, particularly in clinical populations. Chronic inflammation is a hallmark of aging, sarcopenia, obesity, and many chronic diseases, and exercise-induced reductions in inflammatory tone are associated with improvements across these conditions (Cahalin et al., 2022; Li et al., 2025). BFR’s ability to elicit comparable inflammatory adaptations at low mechanical loads makes it particularly attractive for patients who cannot tolerate high-intensity training. In the case of older patients recovering from tibial plateau fractures, conservatively dosed BFR contributed to functional improvements that likely reflect not only muscle and vascular adaptations but also favorable changes in local and systemic inflammatory tone (Cao et al., 2026). The interplay between inflammation, satellite cell function, and muscle regeneration is also notable; macrophages, particularly in their pro-regenerative M2 polarization state, are essential for muscle repair, and BFR-induced cyclical inflammatory pulses may support favorable macrophage polarization patterns. In critically ill survivors, where chronic inflammation contributes to persistent muscle wasting, BFR offers a tool to potentially reset inflammatory tone alongside its more direct effects on satellite cell function and protein turnover (Mathur et al., 2025). The integration of immune adaptations with the broader molecular signature of BFR thus reinforces its position as a uniquely comprehensive intervention (**Figure 6**). Future research should continue to map the temporal and qualitative features of immune adaptation across populations and protocols. The development of standardized immune outcome measures will support more rigorous comparison across studies.

## 9 Neuromuscular and neural adaptations

Recent work has begun to map the neurophysiological responses to BFR exercise more systematically, integrating spinal and corticospinal measures with proposed mechanisms; for a translational synthesis of these neurophysiological responses and mechanisms Fox et al. (2026). Here we discussed the motor unit recruitment, electromyographic activity and pain modulation and central nervous system in detail.

### 9.1 Motor unit recruitment and electromyographic activity

One of the early mechanistic explanations for the surprising efficacy of low-load BFR exercise was that the technique produced disproportionate recruitment of high-threshold motor units, traditionally accessed only through high-load contractions. Surface electromyography studies have consistently shown that BFR resistance exercise elicits greater muscle activation, particularly toward the end of sets, than equivalent low-load exercise without occlusion (Yasuda et al., 2013; He et al., 2025). The proposed mechanism involves accelerated peripheral fatigue of slow-twitch fibers under hypoxic and metabolite-rich conditions, which compels the recruitment of fast-twitch fibers to maintain force output. This recruitment pattern resembles that of high-load resistance training and provides a biophysical foundation for the comparable hypertrophic outcomes observed across modalities. Although surface electromyography has methodological limitations, including signal contamination by motion artifacts and uncertainties about deep fiber activation, complementary approaches including intramuscular electromyography and near-infrared spectroscopy support the conclusion that BFR enhances motor unit recruitment. Importantly, the catecholamine surge associated with BFR exercise also enhances motor unit excitability through adrenergic modulation of motoneuron and muscle fiber properties (Bane et al., 2024). The integrated neural and metabolic stimulus thus reaches motor units that would otherwise remain dormant during low-load exercise. This recruitment pattern is one of the central pillars of BFR’s hypertrophic efficacy.

Beyond fiber recruitment, BFR exercise has been associated with cross-education effects, where strength gains in the trained limb are accompanied by smaller but measurable improvements in the untrained contralateral limb. This phenomenon, well documented in traditional resistance training, is consistent with central nervous system adaptations including changes in corticospinal excitability and motor cortex representation. Recent work by Kataoka et al. found that submaximal low-load BFR resistance exercise produced muscle-size and strength gains similar to low-load exercise performed to failure, although it did not match low-load-to-failure training for muscular endurance (Kataoka et al., 2026). Cross-education strength transfer to the untrained contralateral limb was not an endpoint of that study; rather, cross-education is a separately documented phenomenon in resistance training, consistent with central neural adaptation, and is discussed here in that general context rather than attributed to the above trial. These findings suggest that BFR provides sufficient neural drive to engage central adaptive mechanisms despite its modest mechanical signature. The case of post-surgical patients again provides a clinically relevant illustration; following anterior cruciate ligament reconstruction, patients exhibit profound deficits in voluntary muscle activation, partly attributable to arthrogenic muscle inhibition. BFR has been shown to attenuate these deficits, possibly through a combination of metaboreflex-driven neural facilitation and central adaptation (Solie et al., 2023; Butt and Ahmed, 2024). The neural adaptations to BFR may also play a role in fall prevention in older adults, as improvements in motor unit recruitment and rate of force development are associated with reduced fall risk. Continued investigation into the neural foundations of BFR adaptation will support more targeted protocol development. As motor unit imaging and electrophysiological techniques advance, more refined characterization of these adaptations will be possible.

### 9.2 Pain modulation and central nervous system effects

Pain perception and modulation represent additional dimensions of the central nervous system response to BFR exercise, with implications both for tolerance of the technique and for therapeutic applications in chronic pain conditions. Acute BFR exercise produces sensations of muscle tightness, burning, and discomfort that reflect the activation of group III and IV muscle afferents responsive to metabolic and mechanical stimuli. These sensations are typically rated higher than those of equivalent low-load exercise without occlusion, although they remain tolerable for most participants and resolve rapidly upon cuff release. Importantly, the acute pain response to BFR exercise can engage descending modulatory pathways that produce post-exercise hypoalgesia, a phenomenon associated with reductions in pain sensitivity at sites distant from the trained limb (Hughes et al., 2017; Xie et al., 2025). This hypoalgesic effect has been observed both in healthy adults and in clinical populations with chronic pain, including patients with knee osteoarthritis and chronic low back pain. A recent mini-review by Xie et al. highlighted the mechanisms of BFR for knee pain, including modulation of nociceptive signaling, anti-inflammatory effects, and improvements in muscle function that reduce mechanical load on the affected joint (Xie et al., 2025). The integration of analgesic effects with anabolic adaptations makes BFR particularly attractive for chronic pain populations. The hypoalgesic mechanisms involve both peripheral and central components, including endogenous opioid release and central pain modulation. The combined effect of these mechanisms positions BFR as a unique tool for pain management.

The application of BFR in chronic pain populations has expanded substantially in recent years, with growing evidence supporting its efficacy in conditions ranging from knee osteoarthritis to chronic low back pain and patellofemoral pain syndrome. In a 2026 study by Werasirirat et al., the integration of BFR with core stabilization exercise improved muscle activity and thickness in patients with chronic low back pain, supporting the technique as a useful adjunct in spinal rehabilitation (Werasirirat et al., 2026). The mechanisms underlying these benefits include direct improvements in muscle function, reductions in pain through hypoalgesic pathways, and improvements in patient engagement attributable to the relatively comfortable nature of low-load exercise. In knee osteoarthritis, a substantial body of work has shown that BFR resistance training improves both pain and function compared with conventional low-load training, and the molecular mechanisms identified by Xie et al. provide a mechanistic foundation for these clinical benefits (Xie et al., 2025). The case of older patients with tibial plateau fractures further illustrates the integration of pain modulation with functional rehabilitation, as the conservative BFR protocol improved knee function and quality of life with minimal pain-related dropout (Cao et al., 2026). The pain-modulating effects of BFR are also relevant in athletic contexts, where the technique can support training during periods of injury or pain that would otherwise compromise loading. As chronic pain remains one of the most prevalent and costly conditions globally, the integration of BFR into multidisciplinary pain management programs offers substantial promise. Continued research into the neural and pharmacological mechanisms underlying BFR-induced hypoalgesia will support the development of more targeted applications. The integration of pain neuroscience with exercise physiology in BFR research is a particularly promising direction.

## 10 Translational applications across clinical populations

### 10.1 Post-surgical rehabilitation and orthopedic recovery

Post-surgical orthopedic rehabilitation is among the most extensively studied and clinically validated applications of BFR exercise, with anterior cruciate ligament reconstruction representing the prototypical use case. Following anterior cruciate ligament reconstruction, patients typically experience profound quadriceps atrophy, weakness, and persistent functional deficits that conventional low-load rehabilitation is poorly equipped to reverse owing to the limits imposed by surgical recovery. BFR offers a means of generating high-load equivalent stimuli at low mechanical loads, allowing rehabilitation to progress more rapidly while respecting surgical constraints (Solie et al., 2023; Butt and Ahmed, 2024). The case of a fifteen-year-old male soccer player previously cited illustrates these principles in vivid detail; following anterior cruciate ligament reconstruction with a bone–patellar tendon–bone autograft, he completed a sixteen-week protocol integrating clinic-based BFR with home-based practical BFR using elastic cuffs. By the end of rehabilitation, his surgical limb had achieved larger thigh girth and lean body mass than the contralateral non-surgical limb, an outcome that is exceptional in this clinical context (Solie et al., 2023). A 2024 systematic review by Butt and Ahmed evaluated BFR efficacy in anterior cruciate ligament reconstruction rehabilitation across multiple randomized trials, finding that the majority of studies measuring muscle mass reported significant findings favoring BFR (Butt and Ahmed, 2024). Other meta-analyses have similarly identified favorable effects on knee extensor and flexor isokinetic strength and quadriceps cross-sectional area (Cerqueira et al., 2024). Beyond anterior cruciate ligament reconstruction, BFR has been applied successfully in rehabilitation following meniscectomy, total knee arthroplasty, rotator cuff repair, and Achilles tendon repair. The technique’s ability to support muscle preservation and recovery during periods of restricted loading is one of its most clinically transformative features.

The integration of BFR into orthopedic rehabilitation programs is increasingly supported by structured clinical guidelines, although protocol heterogeneity remains a challenge for evidence synthesis. The 2024 protocol described by Butt and Ahmed identifies key parameters for implementation including the appropriate range of cuff pressures, exercise loads, and timing relative to surgery (Butt and Ahmed, 2024). In acute postoperative phases, where weight bearing and joint loading are restricted, BFR provides one of the few effective means of generating anabolic stimuli to vulnerable muscle groups. As rehabilitation progresses through subacute and return-to-sport phases, BFR can be combined with progressively higher loads to support the transition to sport-specific demands. The case of patients undergoing rehabilitation for tibial plateau fractures, recently described by Cao et al., extends these principles to populations whose recovery is often complicated by age, comorbidities, and prolonged immobilization (Cao et al., 2026). In their cohort, short-term BFR training improved knee function and quality of life, supporting the technique’s broad applicability across orthopedic populations. Particular attention has been paid to safety concerns including deep vein thrombosis, with most studies reporting no increased incidence in carefully screened patients. Standard contraindications for BFR rehabilitation include a history of deep vein thrombosis, pulmonary embolism, coagulation disorders, current anticoagulant therapy, and severe peripheral vascular disease. Within these guidelines, BFR has been integrated into clinic-based, home-based, and hybrid rehabilitation programs with growing success. The combination of clinical efficacy, biological mechanisms, and patient acceptability has made BFR a cornerstone tool in modern orthopedic rehabilitation.

### 10.2 Sarcopenia and geriatric functional decline

Sarcopenia, defined as the progressive loss of skeletal muscle mass, strength, and function with age, represents one of the most pressing public health challenges of an aging global population, and BFR has emerged as a particularly promising intervention. Conventional resistance training, while effective for sarcopenia, requires loads that many older adults cannot tolerate owing to joint pain, cardiovascular limitations, balance impairment, and fear of injury. BFR offers a means of generating comparable adaptations at substantially lower mechanical loads, expanding access to evidence-based muscle interventions for vulnerable older adults (Cahalin et al., 2022; Li et al., 2025). Recent biological mechanism reviews have systematically integrated the existing evidence on BFR for sarcopenia, identifying mTORC1 activation, myostatin suppression, satellite cell expansion, and angiogenesis as the principal molecular foundations for clinical benefit (Li et al., 2025). The 2025 network meta-analysis by Ren et al. further refined these recommendations, identifying low-frequency, low-pressure, and low-intensity BFR regimens as producing the most favorable balance between strength gains and cardiovascular safety in older adults (Ren et al., 2025). Sarcopenia-specific outcomes including grip strength, gait speed, chair rise performance, and SARC-F scores have all been shown to improve following BFR training programs in this population. The integration of BFR into routine geriatric exercise prescription is therefore increasingly supported. As global aging accelerates, scalable interventions that maintain muscle health in older adults will be of growing importance.

The molecular foundation for BFR’s effects in sarcopenia is particularly compelling given the multifactorial nature of age-related muscle loss. Older adults exhibit anabolic resistance, characterized by blunted protein synthetic responses to feeding and exercise, alongside reduced satellite cell pools and elevated baseline inflammation (Cruz-Jentoft et al., 2019; Cahalin et al., 2022). BFR’s ability to amplify anabolic signaling at low loads partially compensates for anabolic resistance, while its effects on satellite cells and inflammation address other dimensions of sarcopenia pathophysiology. The case of older patients recovering from tibial plateau fracture demonstrates how these principles translate into clinical outcomes, with conservative BFR protocols producing functional improvements that support independent living and reduce fall risk (Cao et al., 2026). The 2025 review by Li et al. additionally highlighted the importance of safety monitoring in older adults, including baseline cardiovascular assessment, careful pressure individualization, and gradual progression of training volume (Li et al., 2025). Integration of BFR with nutritional interventions including high-quality protein and vitamin D supplementation may further amplify the technique’s effects on muscle anabolism. Group-based BFR exercise programs in community and assisted living settings have been associated with high adherence and patient satisfaction, supporting feasibility at scale. The technique’s relatively low equipment requirements and scalability also make it suitable for low-resource settings. As populations continue to age, scalable interventions for muscle health will become increasingly valuable. The integration of BFR into national and international sarcopenia management guidelines is therefore a logical next step. Continued evidence accumulation, particularly from large pragmatic trials, will support these guideline development efforts.

### 10.3 Cardiovascular and chronic disease management

The application of BFR exercise to populations with cardiovascular and chronic disease represents both an exciting frontier and a domain of legitimate caution, requiring careful integration of mechanistic understanding with safety considerations. In chronic obstructive pulmonary disease, the recent 2026 systematic review by Alves et al. evaluated BFR training across multiple trials and reported improvements in exercise capacity, muscle strength, and pulmonary function (Alves et al., 2026). The mechanism likely involves a combination of peripheral muscle adaptations, including mitochondrial biogenesis and angiogenesis, alongside reduced ventilatory demand attributable to improved muscle efficiency. Patients with chronic obstructive pulmonary disease often suffer from peripheral muscle dysfunction that contributes substantially to their exercise intolerance, and BFR provides a means of addressing this dysfunction without imposing the high ventilatory loads of traditional aerobic training. In hypertension, the cardiovascular safety profile of BFR has been more controversial, with some authors expressing caution about the elevated blood pressure responses observed during exercise (Suggitt et al., 2024; Cristina-Oliveira et al., 2020). Evidence in hypertensive populations is mixed and should be interpreted cautiously. A relatively small meta-analysis reported broadly neutral effects on resting blood pressure following BFR training in carefully screened hypertensive individuals (Wong et al., 2018); however, the acute exercising state is associated with exaggerated pressor and metaboreflex responses, and several authors caution against unselected use of BFR in cardiovascular disease (Cristina-Oliveira et al., 2020; Suggitt et al., 2024). On balance, BFR in hypertension warrants conservative pressures, blood-pressure monitoring, and individualized clearance rather than routine application. The development of population-specific protocols will be essential to maximize benefits while minimizing risks across the diverse landscape of chronic disease. As mechanistic understanding deepens, application to additional populations is likely to expand.

In neurological rehabilitation, exercise training combined with BFR has been examined in hemiplegic patients, with a randomized clinical trial reporting improvements in muscle mass and lower-extremity function and walking capacity, extending BFR’s potential reach into post-stroke care (Feng et al., 2025). In type 2 diabetes mellitus, BFR offers potential benefits through improvements in muscle glucose uptake, insulin sensitivity, and mitochondrial function, mechanisms supported by its effects on AMPK signaling and capillarization (He et al., 2025; Li et al., 2024), although direct clinical trial evidence in type 2 diabetes remains limited. The molecular mechanisms underlying BFR’s metabolic benefits, including AMP-activated protein kinase activation and improved capillarization, are highly relevant to diabetes management, and emerging clinical trials are beginning to test these applications. In peripheral artery disease, where exercise tolerance is limited by claudication and microvascular dysfunction, BFR’s angiogenic effects could theoretically support functional improvements, although the safety considerations in this population require careful evaluation. In heart failure with preserved ejection fraction, where peripheral muscle dysfunction contributes substantially to exercise intolerance, BFR may offer benefits similar to those observed in chronic obstructive pulmonary disease. Cancer-related fatigue and cancer cachexia represent additional emerging applications, with preliminary evidence suggesting that BFR may attenuate muscle wasting and improve function in oncology patients undergoing treatment. The case of critically ill survivors described by Mathur et al. further extends BFR applications into populations with severe deconditioning and ongoing systemic illness (Mathur et al., 2025), consistent with the broader rationale for BFR as a non-pharmacological countermeasure to muscle wasting in cachectic and load-compromised patients Conceição and Ugrinowitsch, 2019). With respect to cancer, there is currently no evidence that BFR prevents malignancy, and claims to that effect would be unwarranted. The more defensible application is supportive: preliminary work suggests BFR may attenuate muscle wasting and improve function in patients undergoing oncological treatment (cancer-related fatigue and cachexia), where its low mechanical load is advantageous (Conceição and Ugrinowitsch, 2019; Mathur et al., 2025). Any systemic anti-inflammatory or metabolic contribution to cancer outcomes remains hypothetical and requires dedicated trials. The integration of BFR into rehabilitation programs for these diverse populations will require careful protocol development, comprehensive safety monitoring, and continued mechanistic research. As the BFR evidence base expands, the technique’s role as a versatile rehabilitation tool across chronic disease will likely become increasingly central. Continued collaboration between exercise physiologists, clinicians, and patients will be essential to realize this potential. The development of disease-specific BFR guidelines will support evidence-based clinical implementation.

### 10.4 Effects on non-occluded and proximal muscle

A recurring practical question is whether muscle proximal to the cuff such as the gluteals, chest, abdominal, and paraspinal muscles benefits from BFR, given that occlusion is applied distally. Two distinct mechanisms are relevant. First, proximal muscles that are directly recruited during a BFR exercise (for example, the gluteals and trunk during a BFR leg press) receive the usual training stimulus of their own contraction, but they do not experience the local hypoxic, metabolite-rich environment created distal to the cuff; their adaptation is therefore driven by contraction rather than by occlusion per se, and is unlikely to exceed what the same contraction would achieve without a cuff. Second, the systemic endocrine and metabolic responses to distal BFR growth hormone, IGF-1, catecholamines, and lactate, circulate and could in principle provide a modest anabolic background to non-occluded muscle; however, the hormone hypothesis of hypertrophy is contested (Lopes et al., 2022), and any remote effect is likely small relative to local contraction. For axial muscles such as the abdominals and paraspinals, which cannot be occluded, there is currently no validated method to deliver a true occlusion stimulus. The practical approaches are therefore (i) to apply limb BFR during compound or anti-rotation movements that co-recruit the trunk, and (ii) to combine conventional core training with limb-based BFR within the same session. The recent integration of BFR with core-stabilization exercise in chronic low back pain illustrates a feasible hybrid model (Werasirirat et al., 2026), but the benefit to the trunk muscles in that context derives chiefly from their contraction rather than from occlusion. We therefore advise against marketing claims that BFR “targets” proximal or core muscles through occlusion; the honest position is that proximal muscle benefits from co-contraction and, possibly, from a small systemic effect, but not from local occlusion.

## 11 Safety profile and risk stratification

The safety profile of BFR exercise has been a topic of substantial investigation, and the accumulated evidence indicates that, when applied with appropriate screening and dosing, the technique is generally safe across diverse populations. Concerns initially centered on the theoretical risks of deep vein thrombosis, rhabdomyolysis, peripheral nerve injury, and cardiovascular events, although large-scale surveillance data have generally not supported elevated incidence of these complications relative to traditional exercise modalities. A surveillance study of KAATSU practitioners in Japan reported low overall adverse event rates, with the most common events being subcutaneous hemorrhage, paresthesia, and cold feet, all of which were transient and self-limiting. Deep vein thrombosis, the most concerning theoretical complication, has not been shown to be more common with BFR than with conventional exercise in adequately powered surveillance, although standard contraindications including a history of thromboembolic disease and current anticoagulant therapy continue to be observed in clinical practice. Rhabdomyolysis has been reported sporadically, particularly in cases involving deconditioned individuals subjected to high-volume protocols, and emphasizes the importance of gradual progression. Cardiovascular adverse events are theoretically possible owing to the exaggerated hemodynamic responses associated with BFR exercise, and patients with severe cardiovascular disease should undergo individualized risk assessment before participation (Suggitt et al., 2024; Lin et al., 2025). The development of standardized risk stratification tools, building on the work of Patterson et al., has supported safer clinical implementation. As BFR continues to expand into broader clinical and community settings, ongoing safety surveillance will remain essential to monitor for rare adverse events. Continued reporting of adverse events through professional registries will support evidence-based safety guidelines.

The most-cited field-safety data come from a national survey of KAATSU (BFR) practice in Japan. Nakajima et al. surveyed 105 facilities encompassing 12,642 participants and reported low overall adverse-event rates: the most frequent events were subcutaneous hemorrhage/bruising at the cuff site (13.1%) and transient numbness (1.3%), with lightheadedness uncommon (0.3%). Serious events were rare, venous thrombus 0.055%, pulmonary embolism 0.008%, and rhabdomyolysis 0.008%, and the reported venous-thrombosis rate did not exceed background rates in the general population (Nakajima et al., 2006). These surveillance data, together with more recent controlled work, support the view that, with appropriate screening and dosing, BFR is generally safe across diverse populations. Risk stratification should precede prescription. We summarize absolute and relative contraindications in **Table 5** and direct clinicians to the decision pathway in **Figure 4**. Absolute contraindications include active or recent venous thromboembolism, known thrombophilia or current anticoagulation for active thrombosis, and severe peripheral arterial disease; relative contraindications, requiring individualized risk–benefit assessment, include a history of deep-vein thrombosis or pulmonary embolism, uncontrolled hypertension, advanced heart failure, sickle-cell trait/disease, pregnancy, and the presence of vascular grafts or recent vascular surgery in the limb. In higher-risk individuals, lower cuff pressures, shorter exposures, and closer monitoring are prudent starting points.

**Table 5:** Absolute and relative contraindications to BFR exercise.

Absolute contraindications	Relative contraindications (individualized risk–benefit)
Active or recent venous thromboembolism (DVT/PE)	History of DVT or PE (resolved)
Known thrombophilia/current anticoagulation for active thrombosis	Uncontrolled hypertension
Severe peripheral arterial disease	Advanced heart failure
Active limb infection or open wound under the cuff	Sickle-cell trait or disease
Recent vascular surgery or graft in the target limb	Pregnancy
	Severe varicose veins; lymphoedema; significant neuropathy

DVT, deep-vein thrombosis; PE, pulmonary embolism. Lists are indicative and should be applied alongside individualized clinical judgement and the decision pathway in **Figure 4**. Adapted from Patterson et al. (2019); Cristina-Oliveira et al. (2020), and Nakajima et al. (2006).

Several limitations of this review should be acknowledged. First, we did not perform a formal risk-of-bias appraisal or quantitative synthesis, so the relative quality of the underlying studies is not graded uniformly. Second, the search was limited to English-language publications, introducing potential language bias and possibly under-representing relevant work, including from BFR/KAATSU literatures published in other languages. Third, several conclusions rely on recent secondary syntheses (systematic reviews and meta-analyses) rather than on primary data we re-analyzed, and a number of 2025–2026 sources are very recent. Finally, much of the mechanistic literature derives from small, biopsy-based studies in young, healthy participants, which constrains generalizability to the clinical populations discussed. Readers should therefore weight the cross-system integration offered here as a synthesis of current best evidence rather than as a definitive quantitative appraisal.

## 12 Future research directions and conclusion

A central limitation in interpreting the BFR literature and therefore this review is the marked methodological heterogeneity across studies. Cuff width and material, the method of pressure prescription (a percentage of limb occlusion pressure versus fixed absolute or arbitrary pressures), exercise mode, load, repetition scheme, inter-set rest, and total volume all vary widely, as do the populations studied (resistance-trained versus untrained, young versus older, and healthy versus clinical cohorts). Because limb occlusion pressure itself depends on cuff width, limb circumference, and measurement posture, nominally similar pressures can produce substantially different degrees of vascular restriction between studies. This heterogeneity limits direct quantitative comparison, inflates between-study variability, and means that pooled or narrative conclusions should be read as indicating the direction and plausibility of effects rather than precise effect magnitudes. Despite the rapid expansion of BFR research over the past decade, important knowledge gaps remain that will shape the trajectory of the field over the coming years. Personalized prescription based on genetic, molecular, and physiological phenotyping represents one frontier; a 2026 scoping review highlighted the absence of studies linking specific genetic polymorphisms to BFR adaptation, despite the well-established role of genetic variation in exercise responsiveness more broadly. The development of validated biomarkers for predicting individual response to BFR, including markers of mTORC1 sensitivity, satellite cell pool size, and inflammatory tone, will be valuable for tailoring protocols to individual patients. The optimization of BFR for specific clinical populations, including those with cardiovascular disease, diabetes, cancer cachexia, and neurodegenerative disease, requires dedicated trials with appropriate safety monitoring. The integration of BFR with emerging exercise modalities including neuromuscular electrical stimulation, whole-body vibration, and high-intensity interval training has shown promise in preliminary investigations and merits further development. Real-time monitoring technologies, including near-infrared spectroscopy, electromyography, and continuous hemodynamic measurement, may support feedback-driven prescription that maximizes adaptation while minimizing risk. The molecular basis of inter-individual variability in response to BFR also warrants deeper investigation, including the contributions of sex, age, training history, and underlying disease. As the field matures, harmonization of protocols, outcome measures, and reporting standards will support more rigorous evidence synthesis and clinical translation. Continued mechanistic work into the integration of mechanical, metabolic, endocrine, and immune signaling will refine our understanding of how BFR achieves its remarkable effects. The next decade of BFR research is likely to bring substantial advances across all these dimensions. In addition, whether BFR confers cognitive or neuroprotective benefit, for example, in dementia prevention is presently speculative. Aerobic and resistance exercise are associated with reduced dementia risk, and BFR’s ability to elicit exercise-like systemic responses (including possible BDNF involvement) at low mechanical load makes it a plausible candidate for older adults who cannot tolerate higher-intensity exercise. When training load is matched to the individual’s clinical context, low-load BFR can produce hypertrophy and strength gains comparable to high-load resistance training, and may be preferable when high mechanical loads are contraindicated (e.g., early post-surgical rehabilitation, sarcopenia, or selected chronic-disease populations). It is not, on current evidence, uniformly superior to high-load training in healthy, load-tolerant individuals. However, no trial has yet evaluated cognitive endpoints with BFR, so this is offered strictly as a hypothesis-generating future direction rather than a claimed effect.

We highlight four priority areas where progress would most advance the field: (1) adequately powered, head-to-head trials standardizing pressure as %LOP and reporting cuff width and measurement position, to resolve dose–response and heterogeneity questions; (2) direct human tests of the proposed signaling mechanisms (e.g., lactate/lactylation and cell-swelling pathways) linking acute responses to chronic hypertrophy; (3) longer-term safety and efficacy data in clinical and older populations, including cardiovascular and post-surgical cohorts, with standardized adverse-event reporting; and (4) integration of BFR into holistic training-load management, including its systemic stress cost and its optimal placement within periodized programs. Remaining questions, while numerous, are secondary to these priorities.

Blood flow restriction exercise is a versatile training modality whose molecular effects extend beyond the trained muscle to influence endocrine, cardiovascular, immune, and neuromuscular signaling. Although these systemic molecular signals are robust, the acute growth-hormone surge being a clear example the magnitude of systemic clinical adaptation is modest in most populations, and the principal, best-evidenced benefits remain loyal to the exercised musculature. The convergence of mTORC1 activation, myostatin suppression, satellite cell expansion, hypoxia-inducible factor-1 alpha-mediated angiogenesis, and growth hormone surge produces hypertrophic and functional outcomes comparable to high-load resistance training, despite mechanical loads that are a fraction of those traditionally considered necessary for adaptation. The integrated metabolic stress generated by partial arterial inflow restriction and venous occlusion engages reactive oxygen species signaling, AMP-activated protein kinase activation, heat shock protein induction, and cellular swelling, providing a uniquely rich biological stimulus that is poorly replicated by other interventions. Recent investigations published in 2025 and 2026 have refined our understanding of optimal dosing across populations and clarified the mechanistic foundations for clinical applications ranging from anterior cruciate ligament reconstruction to sarcopenia, chronic obstructive pulmonary disease, and intensive care unit-acquired weakness. The strongest evidence that these mechanistic insights translate into clinical benefit comes from systematic reviews and meta-analyses, for example, in ACL-reconstruction rehabilitation (Butt and Ahmed, 2024), in musculoskeletal rehabilitation more broadly (Hughes et al., 2017), and in COPD (Alves et al., 2026). The three cases discussed throughout this review (a young athlete after ACL reconstruction, critically ill survivors of ICU-acquired weakness, and older adults rehabilitating from tibial plateau fractures) are offered as illustrations of clinical potential rather than as generalizable evidence, given that two are single-case or pilot studies. Safety considerations, particularly with respect to cardiovascular and thromboembolic risks, remain important and warrant individualized risk stratification, but the accumulated evidence supports BFR as a generally safe intervention when applied with appropriate screening and dosing. Future research priorities include personalized prescription based on molecular phenotyping, expansion to additional clinical populations, integration with emerging exercise modalities, and harmonization of protocols and outcome measures. The trajectory of BFR research over the past decade has been remarkable, and its continued evolution promises to further reshape how clinicians, scientists, and athletes approach the challenge of generating muscular and systemic adaptation under conditions of reduced mechanical load. As the field matures, BFR is poised to assume an increasingly central role in modern rehabilitation, geriatric care, chronic disease management, and athletic performance optimization. By integrating molecular insight with thoughtful clinical translation, BFR exemplifies the promise of mechanism-driven exercise prescription in the twenty-first century.
